# A genome-wide analysis of the phospholipid: diacylglycerol acyltransferase gene family in *Gossypium*

**DOI:** 10.1186/s12864-019-5728-8

**Published:** 2019-05-22

**Authors:** Xinshan Zang, Xiaoli Geng, Lei Ma, Nuohan Wang, Wenfeng Pei, Man Wu, Jinfa Zhang, Jiwen Yu

**Affiliations:** 1 State Key Laboratory of Cotton Biology, Cotton Institute of the Chinese Academy of Agricultural Sciences, Key Laboratory of Cotton Genetic Improvement, Ministry of Agriculture, Anyang, 455000 Henan China; 2 Department of Plant and Environmental Sciences, New Mexico State University, Las Cruces, New Mexico 88003 USA

**Keywords:** Cotton, Phospholipid: diacylglycerol acyltransferase, Expression pattern, Cottonseed oil

## Abstract

**Background:**

Cotton (*Gossypium* spp.) is the most important natural fiber crop worldwide, and cottonseed oil is its most important byproduct. Phospholipid: diacylglycerol acyltransferase (PDAT) is important in TAG biosynthesis, as it catalyzes the transfer of a fatty acyl moiety from the *sn*-2 position of a phospholipid to the *sn*-3 position of *sn*-1, 2-diacylglyerol to form triacylglycerol (TAG) and a lysophospholipid. However, little is known about the genes encoding PDATs involved in cottonseed oil biosynthesis.

**Results:**

A comprehensive genome-wide analysis of *G. hirsutum*, *G. barbadense*, *G. arboreum*, and *G. raimondii* herein identified 12, 11, 6 and 6 *PDAT*s, respectively. These genes were divided into 3 subfamilies, and a PDAT-like subfamily was identified in comparison with dicotyledonous *Arabidopsis*. All GhPDATs contained one or two LCAT domains at the C-terminus, while most GhPDATs contained a preserved single transmembrane region at the N-terminus. A chromosomal distribution analysis showed that the 12 *GhPDAT* genes in *G. hirsutum* were distributed in 10 chromosomes. However, none of the *GhPDATs* was co-localized with quantitative trait loci (QTL) for cottonseed oil content, suggesting that their sequence variations are not genetically associated with the natural variation in cottonseed oil content. Most *GhPDATs* were expressed during the cottonseed oil accumulation stage. Ectopic expression of *GhPDAT1d* increased *Arabidopsis* seed oil content.

**Conclusions:**

Our comprehensive genome-wide analysis of the cotton *PDAT* gene family provides a foundation for further studies into the use of *PDAT* genes to increase cottonseed oil content through biotechnology.

**Electronic supplementary material:**

The online version of this article (10.1186/s12864-019-5728-8) contains supplementary material, which is available to authorized users.

## Background

Cotton, especially upland cotton, is the world’s most important fiber crop, and oil is extracted from its oil-rich seeds. Indeed, cotton ranks sixth among the world’s oil crops. Cottonseed oil makes up approximately 16% of the seed weight [1], and is the most valuable product derived from cotton seed. Cottonseed oil is typically composed of approximately 26% saturated palmitic acid (C16:0), 15% monounsaturated oleic acid (C18:1), and 58% polyunsaturated linoleic acid (C18:2) [2]. From 1999 to 2009, the world-wide consumption of vegetable oils increased by > 50% [3]. Therefore, research into the molecular mechanisms of oil biosynthesis and the development of new high-seed oil content cotton varieties using classical breeding techniques and biotechnological approaches is becoming increasingly important.

Triacylglycerols (TAGs) are major components of vegetable oils. The 3 pathways of DAG /TAG production with different FA compositions have previously been reviewed [4]. These pathways are de novo DAG/TAG synthesis (Kennedy pathway), acyl editing to provide PC-modified FA for de novo DAG/TAG synthesis, and PC-derived DAG/TAG synthesis. Phospholipid: diacylglycerol acyltransferase (PDAT) in the second pathway catalyzes the transfer of a fatty acyl moiety from the *sn*-2 position of a phospholipid to the *sn*-3 position of *sn*-1, 2-diacylglyerol, thus forming TAG and a lysophospholipid. PDAT enzyme activity was first identified in the use of phospholipids as acyl donors and DAG as an acceptor for TAG biosynthesis in yeast and plants [5].

*Arabidopsis* contains two *PDAT* genes, *AtPDAT1* (At5g13640) and *AtPDAT2* (At3g44830) [6]. No significant differences were found in total acyl composition or TAG content between 17-day-old *AtPDAT*-overexpressing and wild-type (WT) seedlings [6]. Additionally, the fatty acid content and composition of seeds also showed no significant difference in the *pdat* mutant versus WT [7]. However, in 5-week-old developing *Arabidopsis* leaves, the overexpression or knockout of *AtPDAT1* in led to significant changes in fatty acid and TAG synthesis [8]. *AtPDAT2* is highly expressed in seeds, but plays no role in TAG biosynthesis [6, 9]. In castor bean, 3 *PDAT* genes have been identified [10]. The endoplasmic reticulum-located *PDAT1–2* enhances hydroxy fatty acid accumulation in transgenic castor bean plants [11]. In flax (*Linum usitatissimum*), 6 *PDATs* have been identified (*LuPDAT1*, *LuPDAT2*, *LuPDAT3*, *LuPDAT4*, *LuPDAT5*, and *LuPDAT6*) [12]. *LuPDAT1*/*LuPDAT5* and *LuPDAT2*/*LusPDAT4*, but not *LusPDAT3* or *LusPDAT6*, have the unique ability to preferentially channel a-linolenic acid into TAG. Recently, the *PDAT* gene *Lro1* was shown to be responsible for hepatitis C virus core-induced lipid droplet formation in a yeast model system [13]. *PDAT* genes were also found in the unicellular green alga *Chlamydomonas reinhardtii* [14] and the bacterium *Streptomyces coelicolor* [15]. However, no mammalian counterpart has yet been found.

Previously, a genome-wide analysis of eudicots found 6 *PDATs* in *Gossypium raimondii* (two each in clades V, VI, and VII) [16]. To further understand the complexity of *PDATs* and TAG biosynthetic mechanisms in cotton, we performed a comprehensive genome-wide analysis of the *PDAT* gene family in cotton in the present study.

## Results

### Genome-wide identification and phylogenetic tree analysis of *PDAT* genes

Allotetraploid cotton *G. hirsutum* and *G. barbadense* contain two ancestral genomes: the At and Dt subgenomes. To identify all PDAT proteins in *G. hirsutum* (AD1), *G. barbadense* (AD2), and its two diploid ancestors *G. arboreum* (AA genome) and *G. raimondii* (DD genome), we used *Arabidopsis* PDAT protein sequences (AtPDAT1/At5g13640 and AtPDAT2/At3g44830) to query the four reference genomes to screen out candidate PDAT-like proteins in cotton. Combined with the previously identified PDATs from *G. raimondii* [16], 12 deduced PDATs were identified in *G. hirsutum* [17], 11 in *G. barbadense* [18], 6 in *G. arboreum* [19] and 6 in *G. raimondii* [20].

**Fig. 1:**
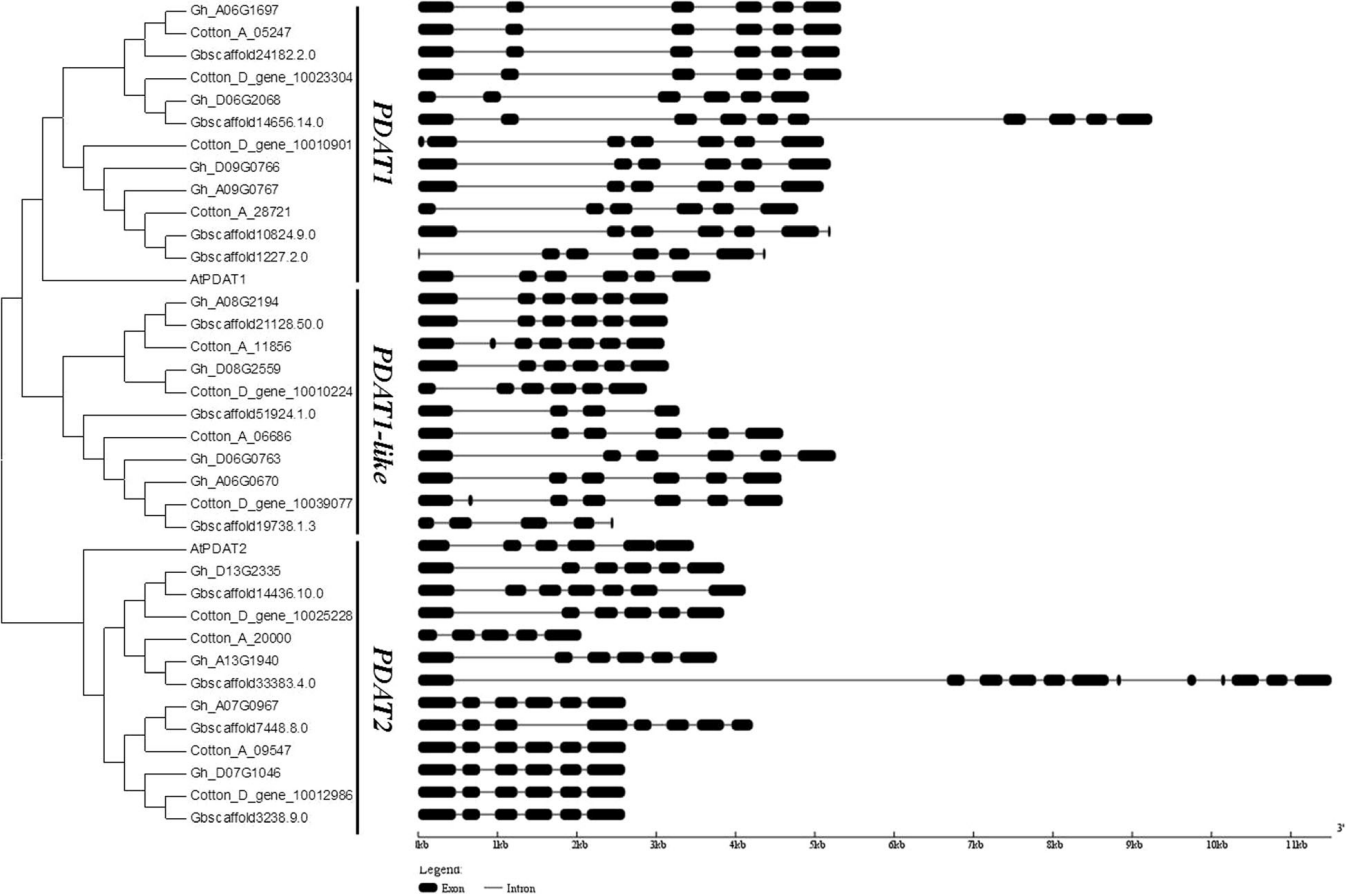
Phylogenetic tree and gene structure of the *PDAT* gene family in *Arabidopsis* and *Gossypium*. The phylogenetic tree of all PDAT proteins in *Arabidopsis* and four *Gossypium* species (Additional file 3) was constructed using Neighbor-Joining method. The exon/intron structure of *PDAT* genes in *Arabidopsis* and four *Gossypium* species. Black boxes show exons and lines show introns

To interpret the relationship between AtPDAT1, AtPDAT2, and cotton PDAT proteins, we constructed a phylogenetic tree (Fig. 1). This classified *PDAT* genes into 3 subfamilies; PDAT1, PDAT1-like, and PDAT2, corresponding to clades VI, V, and VII, respectively [16]. The sequence similarity between GhPDAT1-like and GhPDAT1 was higher than that of GhPDAT2 (Fig. 1). Based on the phylogenetic tree and sequence similarity analysis, we also analyzed orthologous *PDAT* gene pairs in *G. hirsutum*, *G. barbadense*, and their corresponding diploid ancestors (Table 1). Only one gene, *GbPDAT1b*-*like*, was not found or lost in *G. barbadense*. The *PDAT* gene name, gene identifier, gene pairs, and predicted properties of PDAT proteins are listed in Table 1.

**Table 1:** Characteristics of *PDAT* genes and predicted properties of PDAT proteins

Gene name	Gene identifier (NAU)	Chromosomal localization	Size (AA)	MW (KD)	pI
*GaPDAT1a*	Cotton_A_05247	CA_chr12	673	75.5982	8.27
*GhPDAT1a*	Gh_A06G1697	A06	673	75.6993	8.27
*GbPDAT1a*	Gbscaffold24182.2.0	scaffold24182	673	75.6102	8.17
*GrPDAT1b*	Cotton_D_gene_10023304	Chr1	659	73.5794	8.24
*GhPDAT1b*	Gh_D06G2068	D06	598	66.1138	8.45
*GbPDAT1b*	Gbscaffold14656.14.0	scaffold14656	1047	116.7578	8.32
*GaPDAT1c*	Cotton_A_28721	CA_chr11	608	66.9681	6.14
*GhPDAT1c*	Gh_A09G0767	A09	706	78.4205	6.55
*GbPDAT1c*	Gbscaffold1227.2.0	scaffold1227	541	59.6800	5.89
*GrPDAT1d*	Cotton_D_gene_10010901	scaffold121	706	78.3974	6.27
*GhPDAT1d*	Gh_D09G0766	D09	706	78.5186	6.47
*GbPDAT1d*	Gbscaffold10824.9.0	scaffold10824	697	77.3452	6.47
*GaPDAT1a-like*	Cotton_A_11856	CA_chr3	701	78.0008	7.84
*GhPDAT1a-like*	Gh_A08G2194	A08	690	76.9517	7.54
*GbPDAT1a-like*	Gbscaffold21128.50.0	scaffold21128	690	76.9668	7.86
*GrPDAT1b-like*	Cotton_D_gene_10010224	Chr4	598	65.8700	6.90
*GhPDAT1b-like*	Gh_D08G2559	D08	690	76.8657	8.23
*GaPDAT1c-like*	Cotton_A_06686	CA_chr4	705	78.4788	7.09
*GhPDAT1c-like*	Gh_A06G0670	A06	672	74.6619	7.06
*GbPDAT1c-like*	Gbscaffold51924.1.0	scaffold51924	420	46.9796	8.89
*GrPDAT1d-like*	Cotton_D_gene_10039077	Chr10	690	76.6859	6.82
*GhPDAT1d-like*	Gh_D06G0763	D06	672	74.6179	7.06
*GbPDAT1d-like*	Gbscaffold19738.1.3	scaffold19738	370	41.1287	6.52
*GaPDAT2a*	Cotton_A_20000	CA_chr8	532	59.4257	9.00
*GhPDAT2a*	Gh_A13G1940	A13	677	75.6276	8.99
*GbPDAT2a*	Gbscaffold33383.4.0	scaffold33383	1106	123.1350	8.66
*GrPDAT2b*	Cotton_D_gene_10025228	Chr13	677	75.7095	8.89
*GhPDAT2b*	Gh_D13G2335	D13	677	75.5282	8.61
*GbPDAT2b*	Gbscaffold14436.10.0	scaffold14436	695	77.8128	9.04
*GaPDAT2c*	Cotton_A_09547	CA_chr1	691	77.3553	8.41
*GhPDAT2c*	Gh_A07G0967	A07	691	77.3703	8.41
*GbPDAT2c*	Gbscaffold7448.8.0	scaffold7448	1028	115.3379	8.45
*GrPDAT2d*	Cotton_D_gene_10012986	Chr1	691	77.3292	8.10
*GhPDAT2d*	Gh_D07G1046	D07	691	77.2912	8.10
*GbPDAT2d*	Gbscaffold3238.9.0	scaffold3238	691	77.3391	8.10

### Gene structure analysis and chromosomal distribution of *PDAT* genes in cotton

Generic Feature Format files of the four *Gossypium* species were used to analyze the exon-intron structure of putative *PDAT* genes. Figure 1 shows the exon-intron structure of each gene. Although the locations of introns differed, most *PDAT* genes contained 5 introns and 6 exons. For example, in the *PDAT1* subfamily, *AtPDAT1*, *GbPDAT1a* (*Gbscaffold24182.2.0*), and the counterparts from *G. hirsutum*, *G. arboreum* and *G. raimondii* included 5 introns and 6 exons. However, the other 3 *PDAT1* genes *GbPDAT1b* (*Gbscaffold14656.14.0*), *GbPDAT1c* (*Gbscaffold1227.2.0*), and *GbPDAT1d* (*Gbscaffold10824.9.0*), contained 9 introns and 10 exons, 6 introns and 7 exons and 6 introns and 7 exons, respectively. Interestingly, only 3 of 11 *PDAT* genes in *G. barbadense* had the same gene structure.

**Fig. 2:**
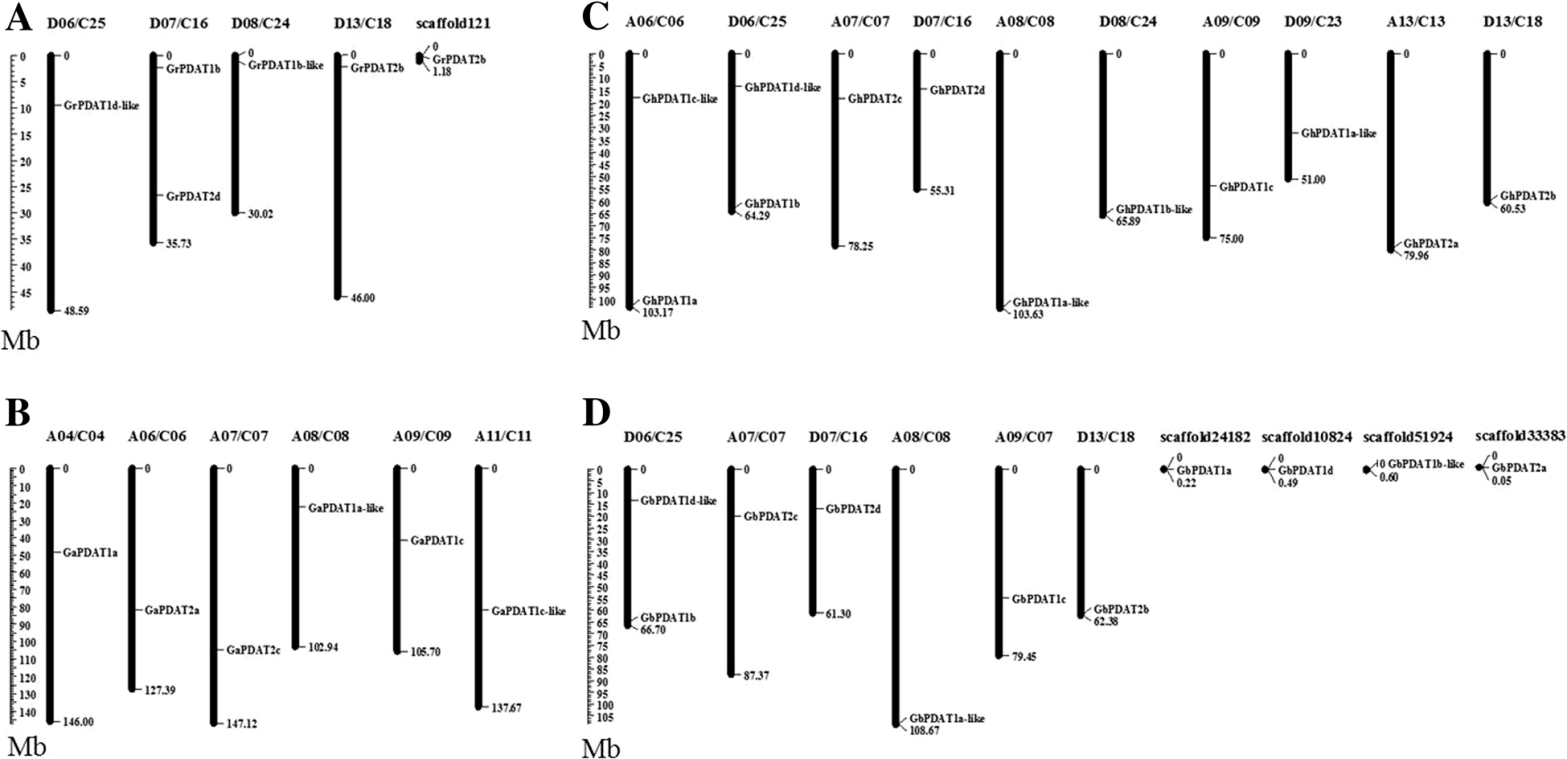
Localization of *PDAT* genes in the four cotton species. Thirty-five *PDAT* genes were mapped on different chromosomes in *Gossypium raimondii* (**a**), *Gossypium arboreum* (**b**), *Gossypium hirsutum* (**c**), and *Gossypium barbadense* (**d**). Only the chromosomes where *PDAT* genes were mapped are shown. The scale represents the megabases (Mb)

Based on the sequenced genome sequence, cotton *PDAT* genes were physically mapped to chromosomes (Fig. 2; Table 1). In *G. hirsutum* and *G. barbadense*, *PDAT* genes were uniformly distributed on the At and Dt chromosome, excluding one lost in *G. barbadense*. In *G. hirsutum*, 12 *PDAT* genes were located on 5 Dt chromosomes (D6, D7, D8, D9 and D13) and 5 At chromosomes (A6, A7, A8, A9 and A13). Two *PDAT* genes were located on both chromosome A6 and D6. Chromosomal localization data are listed in Fig. 2 and Table 1.

### Protein domain analysis of PDATs in *Gossypium hirsutum*

**Fig. 3:**
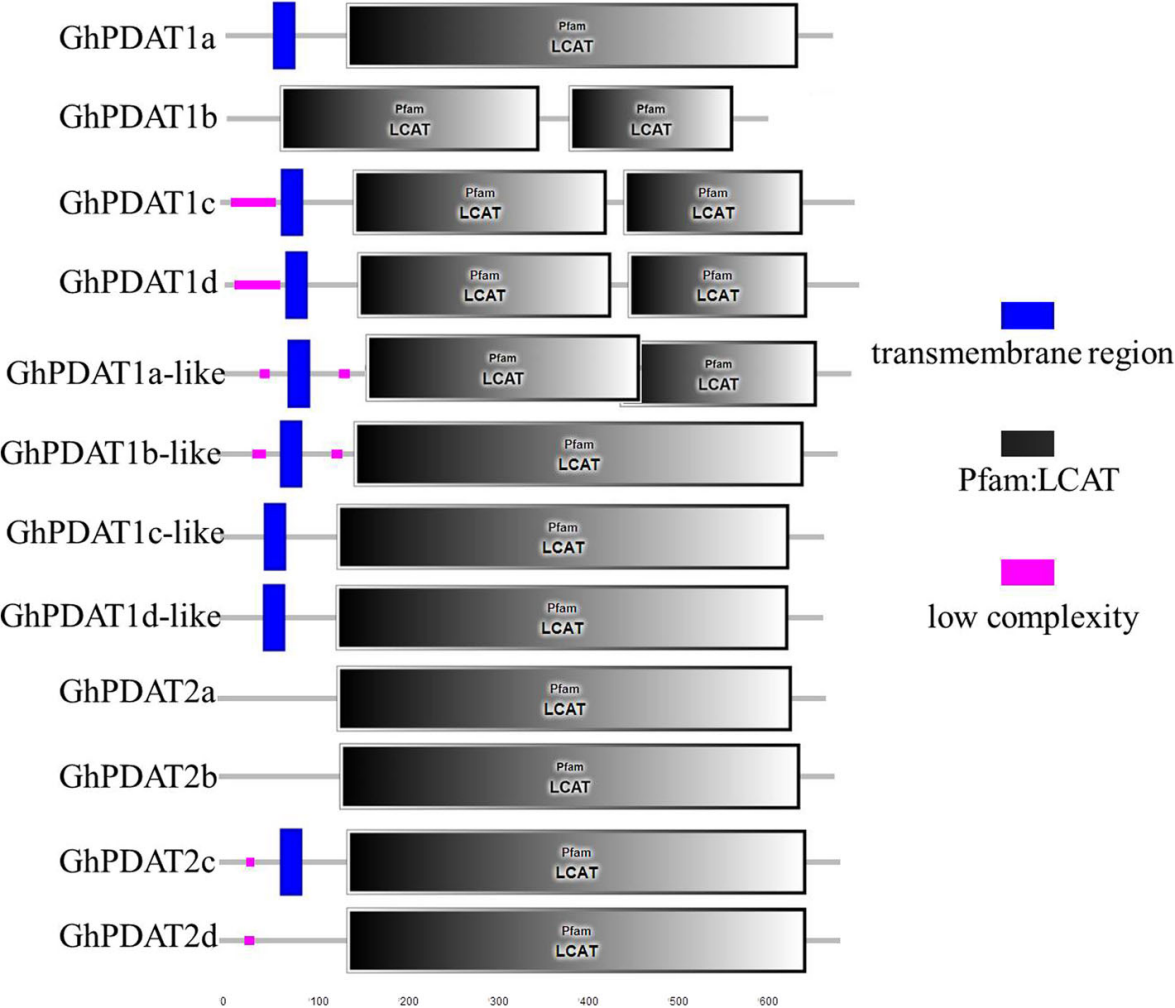
Protein domain prediction for the GhPDATs. The potential transmembrane regions and functional motifs of GhPDAT proteins were identified using SMART database (http://smart.embl-heidelberg.de/)

To improve the comparison of protein domains among GhPDATs, the putative protein domains of 12 GhPDATs were predicted using the SMART database (http://smart.embl-heidelberg.de/). As shown in Fig. 3, a single transmembrane region in the N-terminus has been preserved in most GhPDATs, while all GhPDATs contain one or two LCAT domains at their C-termini.

### Adaptive evolution analysis of the *PDAT* gene family

To explore which type of Darwinian selection determined the process of *PDAT* gene divergence after duplication, the Ka/Ks substitution ratio was used to assess the coding sequences of 12 pairs of *PDAT* gene family orthologs between *G. hirsutum*/*G. barbadense* and *G. arboreum*/ *G. raimondii* (Table 1). A Ka/Ks ratio > 1 represents positive selection, a ratio of 1 represents neutral evolution and a ratio < 1 represents purifying selection [21]. The Ka/Ks ratios of *PDAT* genes ranged from 0.575 to ∞ (Table 2), indicating that the *PDAT* gene family had undergone purifying selection and positive selection in cotton. As shown in Table 2, the majority of *PDAT* genes had undergone positive selection, especially *GbPDAT1b*, *GhPDAT1d*, *GbPDAT1d* and *GhPDAT2d*. Only four *PDAT* genes *GhPDAT1a*, *GbPDAT1a*, *GhPDAT1c* and *GbPDAT2b* had undergone purifying selection.

**Table 2:** Ka and Ks calculations of the orthologous *PDAT* gene pairs

*Gene1*	*Gene2*	Ka	Ks	Ka/Ks
*GaPDAT1a*	*GhPDAT1a*	0.0023	0.004	0.575
*GaPDAT1a*	*GbPDAT1a*	0.0023	0.004	0.575
*GrPDAT1b*	*GhPDAT1b*	2.7289	2.4303	1.123
*GrPDAT1b*	*GbPDAT1b*	0.0129	0.004	3.225
*GaPDAT1c*	*GhPDAT1c*	2.5535	3.9785	0.642
*GaPDAT1c*	*GbPDAT1c*	1.9102	n.a.	n.a.
*GrPDAT1d*	*GhPDAT1d*	0.0023	0	∞
*GrPDAT1d*	*GbPDAT1d*	0.0035	0	∞
*GaPDAT1a-like*	*GhPDAT1a-like*	3.4262	2.9934	1.145
*GaPDAT1a-like*	*GbPDAT1a-like*	3.2594	2.921	1.116
*GrPDAT1b-like*	*GhPDAT1b-like*	2.601	n.a.	n.a.
*GaPDAT1c-like*	*GhPDAT1c-like*	0.7075	0.6125	1.155
*GaPDAT1c-like*	*GbPDAT1c-like*	0.6978	0.6234	1.119
*GrPDAT1d-like*	*GhPDAT1d-like*	0.7557	0.6011	1.257
*GrPDAT1d-like*	*GbPDAT1d-like*	2.6586	2.6203	1.015
*GaPDAT2a*	*GhPDAT2a*	n.a.	4.7056	n.a.
*GaPDAT2a*	*GbPDAT2a*	n.a.	n.a.	n.a.
*GrPDAT2b*	*GhPDAT2b*	0.0082	0.008	1.025
*GrPDAT2b*	*GbPDAT2b*	0.6396	0.674	0.949
*GaPDAT2c*	*GhPDAT2c*	0.0071	0.0038	1.868
*GaPDAT2c*	*GbPDAT2c*	3.4138	n.a.	n.a.
*GrPDAT2d*	*GhPDAT2d*	0.0083	0.0038	2.184
*GrPDAT2d*	*GbPDAT2d*	0.0071	0.0038	1.868

Phylogenetic tree analysis showed that each *AtPDAT* gene corresponded to four *PDAT* genes in tetraploid cotton and two genes in diploid cotton. Therefore, the 12 *GhPDATs* were divided into 6 pair of duplicates, and the Ka/Ks ratio for each pair was calculated (Table 3). All Ka/Ks ratios were < 1, suggesting that the *PDAT* genes from *G. hirsutum* have mainly experienced purifying selection pressure.

**Table 3:** Ka and Ks calculations of the *GhPDAT* gene pairs

*Gene1*	*Gene2*	Ka	Ks	Ka/Ks
*GhPDAT1a*	*GhPDAT1b*	0.0184	0.0638	0.288401
*GhPDAT1c*	*GhPDAT1d*	0.0023	0.0428	0.053738
*GhPDAT1a-like*	*GhPDAT1b-like*	0.003	0.0428	0.070093
*GhPDAT1c-like*	*GhPDAT1d-like*	0.003	0.0329	0.091185
*GhPDAT2a*	*GhPDAT2b*	0.0183	0.0971	0.188465
*GhPDAT2c*	*GhPDAT2d*	0.0053	0.0197	0.269036

### Expression profiles of *PDAT* genes in *Gossypium hirsutum*

**Fig. 4:**
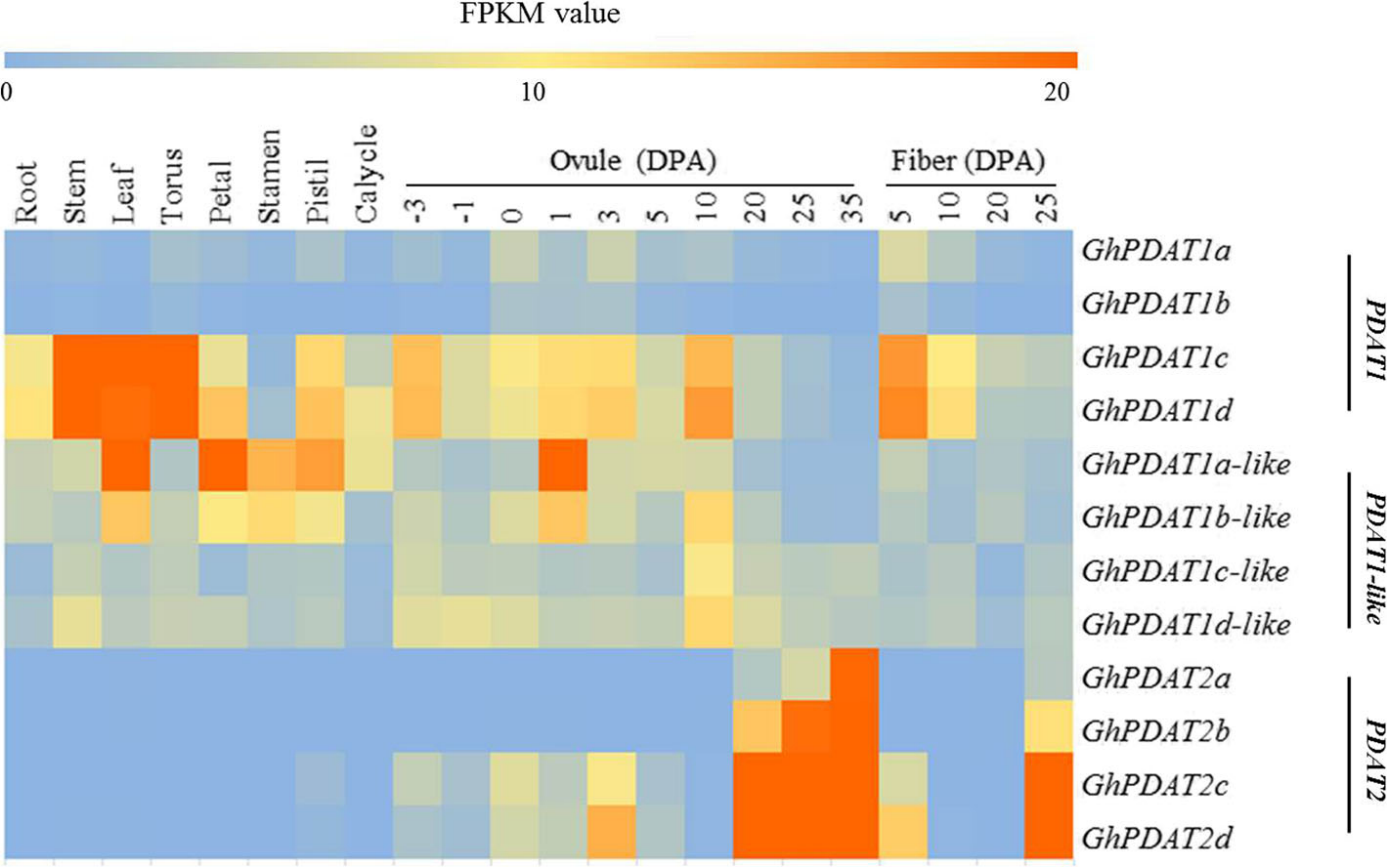
Expression analysis of *GhPDAT* genes in *Gossypium hirsutum* acc TM-1 across 22 tissues. The RNA-seq expression profiles of *G. hirsutum* acc. TM-1 [17] were used to identify the expression levels of *GhPDAT* genes. FPKM represents fragments per kilobase of exon model per million mapped reads

To reveal the gene expression pattern for the *GhPDAT* genes identified, we analyzed the transcript profiles of *PDAT* genes in 22 cotton tissues (Fig. 4) based on published TM-1 data [17]. *GhPDAT1a* and *GhPDAT1b* maintained a low expression level in 22 cotton tissues. *GhPDAT1c* and *GhPDAT1d* were highly expressed in the stem, leaf, and torus, and were also expressed in the ovule and fiber. *GhPDAT1-like* genes were expressed in 22 cotton tissues. *AtPDAT2* was highly expressed in seeds, but plays no role in TAG biosynthesis [6, 9]. *GhPDAT2* was also highly expressed in 20 days post anthesis (DPA)-35 DPA ovules and 25 DPA fibers, and only marginally in other organs. This suggested that *GhPDAT2* plays no role in TAG biosynthesis. Cottonseed oil mainly accumulates in the ovules after 15 DPA-20 DPA, at which stage, most of the *GhPDATs* were expressed. Therefore, *GhPDATs* may play a role in the biosynthesis of TAGs in developing cotton seeds.

To reveal the gene expression pattern for the *GhPDAT* genes identified, we analyzed their transcript profiles in our unpublished RNA-seq datasets. This was based on transcriptomic information for two upland BILs, i.e., 3012 vs. 3008 (with *Gossypium barbadense* germplasm introgression), with differing seed kernel oil contents of 25.88 and 33.52% (Additional file 1: Figure S1). There was no significant difference in the expression levels of *GhPDAT* genes between the two BIL genotypes.

### Co-localization of *PDAT* genes with quantitative trait loci (QTLs) for cottonseed oil

To determine if any *GhPDATs* were genetically associated with the cottonseed oil content, we performed co-localization analysis of *GhPDATs* with QTLs for seed oil content. QTLs were downloaded from the CottonQTL database [22]. However, no *PDAT* gene was localized in the cottonseed oil QTL interval (data not shown).

### Ectopic expression of *GhPDAT1d* increased the oil content of *Arabidopsis* seeds

**Fig. 5:**
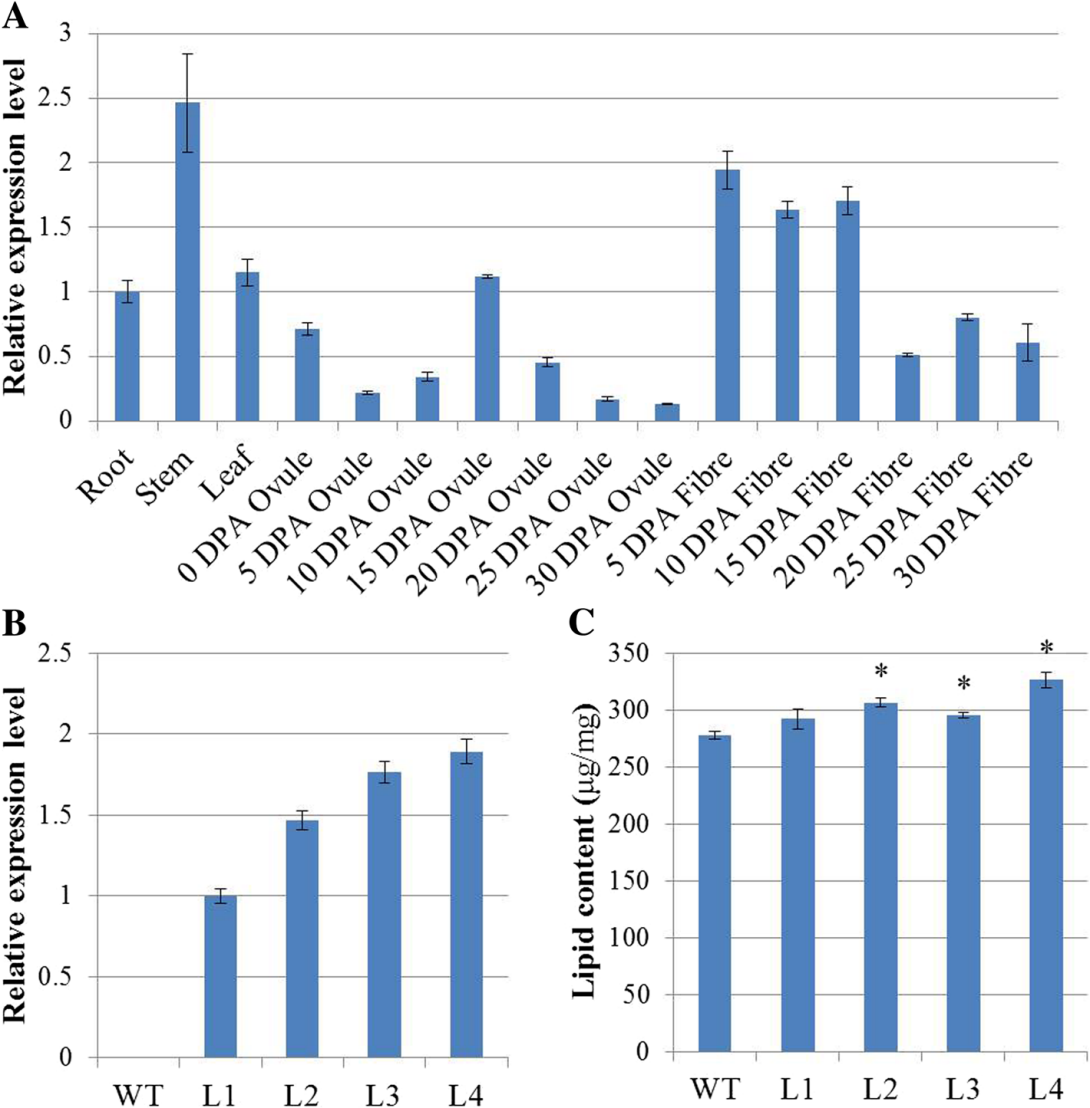
Improved oil content of *GhPDAT1d* transgenic plants. **a** Tissue-specific expression profile of *GhPDAT1d* in different tissues of *G. hirsutum* accession TM-1. The ΔCt value of *GhPDAT1d* in root was set as the control. The data presented are the means ± SD of three replicates. **b** Relative expression level of *GhPDAT1d* in four transgenic *Arabidopsis* lines (L1, L2, L3, and L4). The ΔCt value of *GhPDAT1d* in transgenic line L1 was set as the control. The data presented are the means ± SD of three replicates. **c** Seed oil content of *GhPDAT1d* transgenic lines (L1, L2, L3, and L4) and WT. The data presented are the means ± SD of three replicates; *, *P* < 0.05 (Student’s *t*-test)

In PDAT1 clade, the expression level of *GhPDAT1c* and *GhPDAT1d* (gene pairs from the corresponding At and Dt subgenome) was higher in 15–20 DPA ovules than that of *GhPDAT1a* and *GhPDAT1b* (Figs. 4 and 5a). *GhPDAT1d* was thus selected for further functional analysis. Transgenic *Arabidopsis* plants overexpressing *GhPDAT1d* were generated and used to characterize its biological functions in oil content. Relative expression levels of *GhPDAT1d* analyzed by qRT-PCR in transgenic *Arabidopsis* and WT plants showed that *GhPDAT1d* was highly expressed in the transgenic plants (Fig. 5b). No visible difference between transgenic *Arabidopsis* and WT plants was observed at different developmental stages (data not shown).

In order to determine whether *GhPDAT1d* could increase the oil content, the oil contents of transgenic and WT plants were compared using an NMI20-Analyst nuclear magnetic resonance spectrometer (Niumag, Shanghai, China). Significantly increased oil content, 6.55 to 17.61% higher, was observed in transgenic line L2-L4 (Fig. 5c). There is no significant change in fatty acid compositions of WT and *GhPDAT1d* transgenic Arabidopsis seeds (Table 4).

**Table 4:** Fatty acid compositions of WT and *GhPDAT1d* transgenic *Arabidopsis* seeds. Data are averages of four replicates

	Total lipids (mol %)				
	WT	L1	L2	L3	L4
16:0	8.58 ± 0.143	8.43 ± 0.122	8.36 ± 0.113	8.70 ± 0.145	8.35 ± 0.18
16:1	0.45 ± 0.035	0.42 ± 0.024	0.39 ± 0.025	0.45 ± 0.011	0.39 ± 0.034
18:0	2.72 ± 0.121	2.83 ± 0.127	2.89 ± 0.107	2.69 ± 0.023	2.85 ± 0.075
18:1	11.18 ± 0.261	11.24 ± 0.275	11.63 ± 0.237	11.14 ± 0.019	11.46 ± 0.361
18:2	30.37 ± 0.351	29.43 ± 0.168	29.71 ± 0.161	30.66 ± 0.247	29.49 ± 0.637
18:3	19.54 ± 0.586	19.98 ± 0.232	19.32 ± 0.324	18.44 ± 0.65	19.4 ± 0.241
20:0	2.50 ± 0.010	2.54 ± 0.120	2.48 ± 0.054	2.55 ± 0.027	2.48 ± 0.059
20:1	18.81 ± 0.174	19.22 ± 0.228	19.37 ± 0.113	19.11 ± 0.101	19.64 ± 0.366
20:2	2.37 ± 0.071	2.36 ± 0.027	2.36 ± 0.050	2.59 ± 0.071	2.39 ± 0.081
20:3	0.81 ± 0.012	0.85 ± 0.074	0.81 ± 0.063	0.8 ± 0.021	0.83 ± 0.049
22:0	0.38 ± 0.019	0.38 ± 0.007	0.38 ± 0.009	0.4 ± 0.024	0.36 ± 0.011
22:1	2.29 ± 0.066	2.31 ± 0.026	2.3 ± 0.090	2.46 ± 0.251	2.36 ± 0.106

## Discussion

Despite the fact that many previous studies have revealed a crucial role for *PDAT* encoded products in TAG biosynthesis, our knowledge of *PDATs* in cotton remains limited. Therefore, this study aimed to present an overall picture of *Gossypium PDATs*, including their sequence variation, adaptive evolutionary analysis, protein domains, expression profiles and co-localization with QTLs.

### The *PDAT* gene family in *Gossypium*

*PDAT* genes exist in all plants, including algae, lowland plants (mosses and lycophytes) and highland plants (monocots and eudicots) [16]. This study revealed the details of 12 deduced PDATs from *G. hirsutum*, 11 deduced PDATs from *G. barbadense*, 6 deduced PDATs in *G. arboretum* and 6 deduced PDATs in *G. raimondii*. Evolutionary analysis previously showed that the *PDAT* gene family can be clearly divided into 7 major clades [16]. In the present study, *Gossypium* PDAT amino acid sequences were clustered into 3 clades (subfamilies), and the additional clade, PDAT1-like, was found in cotton. Clades I-IV were not found in cotton. This compares with *Arabidopsis*, in which only two *PDAT* genes (*AtPDAT1* and *AtPDAT2*) have been identified [6].

We observed that each *AtPDAT* gene corresponded to four *PDAT* genes in tetraploid cotton and two genes in diploid cotton. This suggested that *PDAT* gene duplication events occurred in diploid cotton before the emergence of tetraploid cotton, which is consistent with a previously reported eudicot-wide *PDAT* gene expansion [16]. Additionally, a single transmembrane region in the N-terminus has been preserved in most GhPDATs, and one or two LCAT domains were located at the C-terminus of all GhPDATs.

### *PDATs* in relation to seed oil content

Cottonseed oil accumulates in ovules after 15–20 DPA. At this stage, most of the *GhPDATs* were expressed (Fig. 3), indicating that they play a role in the biosynthesis of TAGs in developing cotton seeds. Additionally, we found *GhPDATs* were expressed in developing fibers (Fig. 3), suggesting they are also involved in this stage of development. However, no *PDAT* gene was localized in the cottonseed oil QTL interval (data not shown).

In 5-week-old developing *Arabidopsis* leaves, the overexpression or knockout of *AtPDAT1* led to significant changes in fatty acid and TAG synthesis [8]. Cottonseed oil was widely believed to accumulate in ovules after 15 DPA. At this stage, most *GhPDATs* were found to be expressed (Fig. 4). In this study, we proved that ectopic expression of *GhPDAT1d* could increase the oil content of *Arabidopsis* seeds. Any fatty acid in the seed oil was found to be significantly changed as previously reported *Arabidopsis pdat-ko* mutant [7]. Together, these results implied that *PDATs* are conserved in upland cotton cultivars.

## Conclusion

In conclusion, we performed a comprehensive genome-wide analysis of the *PDAT* gene family in cotton. A total of 35 *PDAT* genes were identified in four sequenced *Gossypium* species and grouped into 3 distinct clades. Ectopic expression of *GhPDAT1d* increased *Arabidopsis* seed oil content. Our detailed analysis of sequence variation, adaptive evolutionary analysis, protein domains, expression profiles, and QTL co-localization provides an important lead for further studies of *PDAT* genes in cotton.

## Methods

### Sequence retrieval, multiple sequence alignment, and phylogenetic analysis

The cotton genome sequences of *G. arboreum* (A2, BGI_V1.0) [19], *G. raimondii* (D5, BGI_V1.0) [20], *G. hirsutum* (AD1, NBI_V1.1) [17] and *G. barbadense* (AD2, SGI_V1.0) [18] were downloaded from the CottonGen database (https://www.cottongen.org). *AtPDAT1* (At5g13640) and *AtPDAT2* (At3g44830) were acquired from TAIR 10 (http://www.arabidopsis.org). To identify *PDAT* genes, *AthPDAT1* and *AthPDAT2* protein sequences were used as queries against cotton genome sequences. Multiple sequence alignments of all identified PDATs in this study were performed using Clustal X2 (http://www.clustal.org/). A phylogenetic tree was constructed using the neighbor-joining algorithm with default parameters and 1000 bootstrap replicates in MEGA 6 (http://www.megasoftware.net/). The sequence length, molecular weight, and isoelectric point of PDAT proteins were calculated using ExPasy (http://web.expasy.org).

### In-silico mapping and genetic structure analysis of *PDAT* genes

Mapping of *PDAT* genes was performed using MapChart (https://www.wur.nl/en/show/Mapchart.htm) [23]. QTLs in this paper were downloaded from CottonQTLdb (http://www.cottonqtldb.org) [22]. The structures of *PDAT* genes were generated using the GSDS (Gene Structure Display Server) algorithm (http://gsds.cbi.pku.edu.cn/).

### Detection of protein domains

Potential transmembrane regions and functional motifs of GhPDAT proteins were identified using the SMART database (http://smart.embl-heidelberg.de/).

### Ka and Ks calculations

*PDAT* gene pairs were used to calculate Ka and Ks using the DnaSP software of phylogenetic analysis by the maximum likelihood method.

### Analysis of *PDAT* genes in RNA-seq data

RNA-seq data of 22 cotton tissues were previously published (accession codes, SRA: PRJNA248163) [17]. Unpublished RNA-seq datasets were generated in our own laboratory using transcriptomic information for two upland BILs, i.e., 3012 vs. 3008 (with *Gossypium barbadense* germplasm introgression), with differing seed kernel oil contents of 25.88 and 33.52%. The expression of *PDAT* genes was analyzed based on these data.

### Transgenic plant generation and expression analysis

Transgenic plant generation and expression analysis were performed as previously reported [24]. Briefly, complete coding sequence of *GhPDAT1d* (Additional file 4) was amplified with gene specific primers from *G. hirsutum* acc. TM-1. The resulting PCR product was cloned into a digested pBI121 vector with BamH I and Sac I using ClonExpress R II One Step Cloning Kit (Vazyme, Nanjing, China). *Agrobacterium tumefaciens* strain *GV3101* containing the binary construct was used to transform *Arabidopsis* plants. We performed quantitative real-time PCR (qRT-PCR) to determine the expression pattern of *GhPDAT1d*, with t2^-ΔΔCt^ method used to quantify the expression level of *GhPDAT1d* relative to the 18S rRNA endogenous control. Primers are listed in Additional file 2: Table S1.

### Oil content analysis

Total oil content was determined with about 0.3 g seeds per sample using an NMI20-Analyst nuclear magnetic resonance spectrometer (Niumag, Shanghai, China) as previously reported [24].

### Fatty acid composition analysis

A gas chromatography/mass spectrometry GC/MS analysis was performed to determine the fatty acid compsitions using a gas chromatograph (7890A, Agilent Technologies, USA) equipped with a flame ionization detector (FID) and an HP-FFAP capillary column (30 m × 250 μm × 0.25 μm). WT and *GhPDAT1d* transgenic *Arabidopsis* seeds (about 100 seeds) were performed to determine the fatty acid components.

## Additional files

Additional file 1: **Figure S1.** Expression analysis of *GhPDAT* genes in our unpublished RNA-seq datasets: with transcriptomic information for two Upland BILs, i.e., 3012 vs. 3008 (with *Gossypium barbadense* germplasm introgression), with differing seed kernel oil content 25.88 and 33.52%. FPKM represents fragments per kilobase of exon model per million mapped reads. (JPG 286 kb)

Additional file 2: **Table S1.** Primers used in this paper. (DOCX 18 kb)

Additional file 3: Phylogenetic data of Fig. 1. (DOCX 21 kb)

Additional file 4: Coding sequence of *GhPDAT1d*. (DOCX 16 kb)

## Abbreviations

DAG: Diacylglycerol
DPA: Days post anthesis
PDAT: Phospholipid: diacylglycerol acyltransferase
QTL: Quantitative trait loci
TAG: Triacylglycerol
WT: Wild-type

## References

[1] Liu Q, Surinder S, Chapman K, Green A (2009). Bridging traditional and molecular genetics in modifying cottonseed oil.

[2] Liu Q, Singh SP, Green AG (2002). High-stearic and high-oleic cottonseed oils produced by hairpin RNA-mediated post-transcriptional gene silencing. Plant Physiol.

[3] Lu C, Napier JA, Clemente TE, Cahoon EB (2011). New frontiers in oilseed biotechnology: meeting the global demand for vegetable oils for food, feed, biofuel, and industrial applications. Curr Opin Biotechnol.

[4] Bates PD, Browse J (2012). The significance of different diacylgycerol synthesis pathways on plant oil composition and bioengineering. Front Plant Sci.

[5] Dahlqvist A, Stahl U, Lenman M, Banas A, Lee M, Sandager L, Ronne H, Stymne S (2000). Phospholipid:diacylglycerol acyltransferase: an enzyme that catalyzes the acyl-CoA-independent formation of triacylglycerol in yeast and plants. Proc Natl Acad Sci U S A.

[6] Stahl U, Carlsson AS, Lenman M, Dahlqvist A, Huang BQ, Banas W, Banas A, Stymne S (2004). Cloning and functional characterization of a phospholipid : diacylglycerol acyltransferase from Arabidopsis. Plant Physiol.

[7] Mhaske V, Beldjilali K, Ohlrogge J, Pollard M (2005). Isolation and characterization of an Arabidopsis thaliana knockout line for phospholipid: diacylglycerol transacylase gene (At5g13640). Plant Physiol Biochem.

[8] Fan J, Yan C, Zhang X, Xu C (2013). Dual role for phospholipid:diacylglycerol acyltransferase: enhancing fatty acid synthesis and diverting fatty acids from membrane lipids to triacylglycerol in Arabidopsis leaves. Plant Cell.

[9] Zhang M, Fan JL, Taylor DC, Ohlrogge JB (2009). DGAT1 and PDAT1 acyltransferases have overlapping functions in Arabidopsis triacylglycerol biosynthesis and are essential for Normal pollen and seed development. Plant Cell.

[10] van Erp H, Bates PD, Burgal J, Shockey J, Browse J (2011). Castor phospholipid:diacylglycerol acyltransferase facilitates efficient metabolism of hydroxy fatty acids in transgenic Arabidopsis. Plant Physiol.

[11] Kim HU, Lee KR, Go YS, Jung JH, Suh MC, Kim JB (2011). Endoplasmic reticulum-located PDAT1-2 from castor bean enhances hydroxy fatty acid accumulation in transgenic plants. Plant Cell Physiol.

[12] Pan X, Siloto RMP, Wickramarathna AD, Mietkiewska E, Weselake RJ (2013). Identification of a pair of phospholipid: diacylglycerol acyltransferases from developing flax (Linum usitatissimum L.) seed catalyzing the selective production of Trilinolenin. J Biol Chem.

[13] Iwasa S, Sato N, Wang CW, Cheng YH, Irokawa H, Hwang GW, Naganuma A, Kuge S (2016). The phospholipid:diacylglycerol acyltransferase Lro1 is responsible for hepatitis C virus core-induced lipid droplet formation in a yeast model system. PLoS One.

[14] Yoon K, Han D, Li Y, Sommerfeld M, Hu Q (2012). Phospholipid: diacylglycerol acyltransferase is a multifunctional enzyme involved in membrane lipid turnover and degradation while synthesizing triacylglycerol in the unicellular green microalga Chlamydomonas reinhardtii. Plant Cell.

[15] Arabolaza A, Rodriguez E, Altabe S, Alvarez H, Gramajo H (2008). Multiple pathways for triacylglycerol biosynthesis in Streptomyces coelicolor. Appl Environ Microbiol.

[16] Pan X, Peng FY, Weselake RJ (2015). Genome-wide analysis of PHOSPHOLIPID:DIACYLGLYCEROL ACYLTRANSFERASE (PDAT) genes in plants reveals the eudicot-wide PDAT gene expansion and altered selective pressures acting on the core eudicot PDAT paralogs. Plant Physiol.

[17] Zhang TZ, Hu Y, Jiang WK, Fang L, Guan XY, Chen JD, Zhang JB, Saski CA, Scheffler BE, Stelly DM (2015). Sequencing of allotetraploid cotton (Gossypium hirsutum L. acc. TM-1) provides a resource for fiber improvement. Nat Biotechnol.

[18] Yuan D, Tang Z, Wang M, Gao W, Tu L, Jin X, Chen L, He Y, Zhang L, Zhu L (2015). The genome sequence of Sea-Island cotton (Gossypium barbadense) provides insights into the allopolyploidization and development of superior spinnable fibres. Sci Rep.

[19] Li FG, Fan GY, Wang KB, Sun FM, Yuan YL, Song GL, Li Q, Ma ZY, Lu CR, Zou CS (2014). Genome sequence of the cultivated cotton Gossypium arboreum. Nat Genet.

[20] Wang KB, Wang ZW, Li FG, Ye WW, Wang JY, Song GL, Yue Z, Cong L, Shang HH, Zhu SL (2012). The draft genome of a diploid cotton Gossypium raimondii. Nat Genet.

[21] Akhunov ED, Sehgal S, Liang HQ, Wang SC, Akhunova AR, Kaur G, Li WL, Forrest KL, See D, Simkova H (2013). Comparative analysis of syntenic genes in grass genomes reveals accelerated rates of gene structure and coding sequence evolution in polyploid wheat. Plant Physiol.

[22] Said JI, Knapka JA, Song MZ, Zhang JF (2015). Cotton QTLdb: a cotton QTL database for QTL analysis, visualization, and comparison between Gossypium hirsutum and G-hirsutum x G-barbadense populations. Mol Gen Genomics.

[23] Voorrips RE (2002). MapChart: software for the graphical presentation of linkage maps and QTLs. J Hered.

[24] Zang XS, Pei WF, Wu M, Geng YH, Wang NH, Liu GY, Ma JJ, Li D, Cui YP, Li XL (2018). Genome-scale analysis of the WRI-like family in Gossypium and functional characterization of GhWRI1a controlling triacylglycerol content. Front Plant Sci.

