# Supplementary figures and images for: A genome-wide analysis of the phospholipid: diacylglycerol acyltransferase gene family in Gossypium
Source: BMC Genomics. 2019 May 22;20:402. doi: 10.1186/s12864-019-5728-8 (PMC6530137; doi:10.1186/s12864-019-5728-8)

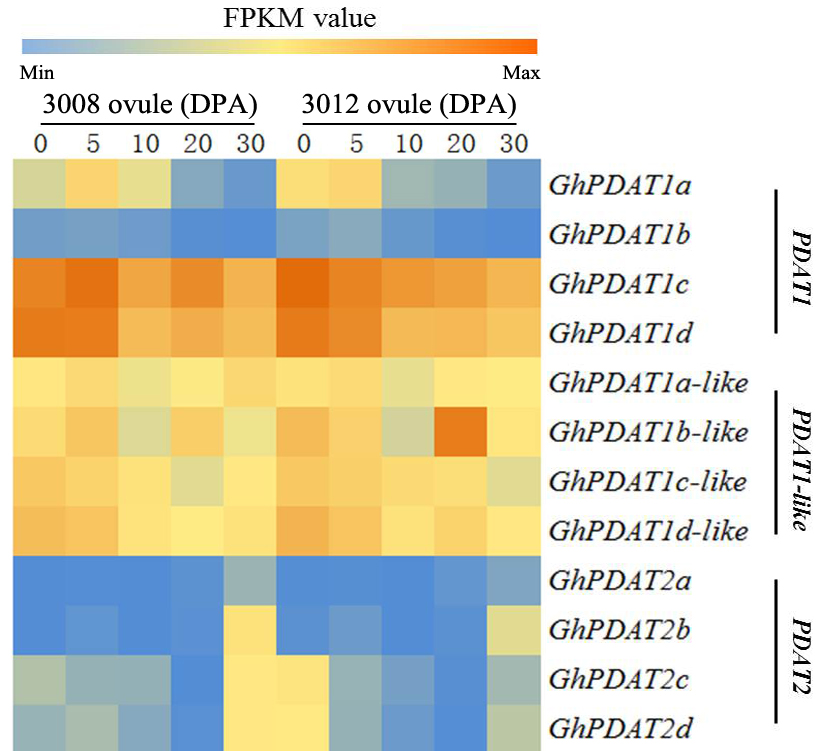

Supplement: Supplementary file 1 — Figure S1. Expression analysis of GhPDAT genes in our unpublished RNA-seq datasets: with transcriptomic information for two Upland BILs, i.e., 3012 vs. 3008 (with Gossypium barbadense germplasm introgression), with differing seed kernel oil content 25.88 and 33.52%. FPKM represents fragments per kilobase of exon model per million mapped reads. (JPG 286 kb) [file 12864_2019_5728_MOESM1_ESM.jpg]
