# Supplementary material for: A genome-wide analysis of the phospholipid: diacylglycerol acyltransferase gene family in Gossypium
Source: BMC Genomics. 2019 May 22;20:402. doi: 10.1186/s12864-019-5728-8 (PMC6530137; doi:10.1186/s12864-019-5728-8)
Supplement: Supplementary file 2 — Table S1. Primers used in this paper. (DOCX 18 kb) [file 12864_2019_5728_MOESM2_ESM.docx]

**Additional file 2: Table S1.** Primers used in this paper.

| **Number** | **Name** | **Sequence (5′-3′)** |
| --- | --- | --- |
| 1 | *GhPDAT1d*-L | ATGTCTTCACTTAGAAGAAGAA |
| 2 | *GhPDAT1d*-R | TTATATGGATATGGATACTTC |
| 3 | Q-*GhPDAT1d*-L | AGAAGAAAACCCATAAATGAAT |
| 4 | Q-*GhPDAT1d*-R | TTGTAAAGGAACAAGAGGAGC |
| 5 | Infusion-*GhPDAT1d*-L | GGACTCTAGAGGATCCATGTCTTCACTTAGAAGAAGAA |
| 6 | Infusion-*GhPDAT1d*-R | GATCGGGGAAATTCGAGCTCTTATATGGATATGGATACTTC |
| 7 | *GhHis3*-L | GAAGCCTCATCGATACCGTC |
| 8 | *GhHis3*-R | CTACCACTACCATCATGGC |
| 9 | *18S rRNA*-L | *ACATCCAAGGAAGGCAGCA* |
| 10 | *18S rRNA*-R | *TAAGACCAGGAGCGTATCGC* |
