# Supplementary material for: A genome-wide analysis of the phospholipid: diacylglycerol acyltransferase gene family in Gossypium
Source: BMC Genomics. 2019 May 22;20:402. doi: 10.1186/s12864-019-5728-8 (PMC6530137; doi:10.1186/s12864-019-5728-8)
Supplement: Supplementary file 3 — Phylogenetic data of Fig. 1. (DOCX 21 kb) [file 12864_2019_5728_MOESM3_ESM.docx]

**Additional file 3.** Phylogenetic data of Figure 1.

>AtPDAT1

MPLIHRKKPTEKPSTPPSEEVVHDEDSQKKPHESSKSHHKKSNGGGKWSCIDSCCWFIGCVCVTWWFLLFLYNAMPASFPQYVTERITGPLPDPPGVKLKKEGLKAKHPVVFIPGIVTGGLELWEGKQCADGLFRKRLWGGTFGEVYKRPLCWVEHMSLDNETGLDPAGIRVRAVSGLVAADYFAPGYFVWAVLIANLAHIGYEEKNMYMAAYDWRLSFQNTEVRDQTLSRMKSNIELMVSTNGGKKAVIVPHSMGVLYFLHFMKWVEAPAPLGGGGGPDWCAKYIKAVMNIGGPFLGVPKAVAGLFSAEAKDVAVARAIAPGFLDTDIFRLQTLQHVMRMTRTWDSTMSMLPKGGDTIWGGLDWSPEKGHTCCGKKQKNNETCGEAGENGVSKKSPVNYGRMISFGKEVAEAAPSEINNIDFRGAVKGQSIPNHTCRDVWTEYHDMGIAGIKAIAEYKVYTAGEAIDLLHYVAPKMMARGAAHFSYGIADDLDDTKYQDPKYWSNPLETKLPNAPEMEIYSLYGVGIPTERAYVYKLNQSPDSCIPFQIFTSAHEEDEDSCLKAGVYNVDGDETVPVLSAGYMCAKAWRGKTRFNPSGIKTYIREYNHSPPANLLEGRGTQSGAHVDIMGNFALIEDIMRVAAGGNGSDIGHDQVHSGIFEWSERIDLKL

>AtPDAT2

MSPLLRFRKLSSFSEDTINPKPKQSATVEKPKRRRSGRCSCVDSCCWLIGYLCTAWWLLLFLYHSVPVPAMLQAPESPGTRLSRDGVKAFHPVILVPGIVTGGLELWEGRPCAEGLFRKRLWGASFSEILRRPLCWLEHLSLDSETGLDPSGIRVRAVPGLVAADYFAPCYFAWAVLIENLAKIGYEGKNLHMASYDWRLSFHNTEVRDQSLSRLKSKIELMYATNGFKKVVVVPHSMGAIYFLHFLKWVETPLPDGGGGGGPGWCAKHIKSVVNIGPAFLGVPKAVSNLLSAEGKDIAYARSLAPGLLDSELLKLQTLEHLMRMSHSWDSIVSLLPKGGEAIWGDLDSHAEEGLNCIYSKRKSSQLSLSNLHKQNYSLKPVSRVKEPAKYGRIVSFGKRASELPSSQLSTLNVKELSRVDGNSNDSTSCGEFWSEYNEMSRESIVKVAENTAYTATTVLDLLRFIAPKMMRRAEAHFSHGIADDLDDPKYGHYKYWSNPLETKLPEAPEMEMYCLYGVGIPTERSYIYKLATSSGKCKSSIPFRIDGSLDGDDVCLKGGTRFADGDESVPVISAGFMCAKGWRGKTRFNPSGMDTFLREYKHKPPGSLLESRGTESGAHVDIMGNVGLIEDVLRIAAGASGQEIGGDRIYSDVMRMSERISIKL

>Gh_A06G1697

MSLLRRRKHPFNESFRKPEKEHENGDFSYESKRKKIAYEHEEERPLSKWSCFDTWCWLIGCVCVTWWFILFLYNAMPASFPKYVTEVITGPLPDPPGVKLRKLGLTAKHPVVFVPAAVTGGLELWEGRKCAQGLFRKRLWGGTYGDVYKRPQCWVEHMSLDNETGMDPCGIRVRPISGLVTADGFGSGCFVWTTLVSNLARIGYEEKTMFMAAYDWRLSFQNTQVRDQTLSRIKNNIELMVATSGGQKVVIIPHSTGALFFLHFMKWVEAPPPMGGGGGPYWCSKHIKAVVNIAAPFLGVPKAIPLFLSAESKDISLVRAIAPGFFENDIFQPQILQHVMRLSHSWDSTVSMIPRGGDTIWGGLDWSPEEGYSCDQKREMKNSTLVTNPARAKSAISLTRSANFGRIVSFGKDVAEAPSNDIDRIDFKGAVKGHRAENRTCRDLWTEYHGMGFEGIKAFADYKTYTAESIVELLHFVAPKMMTRSTAHFSYGIADNMDDPKYKHYKYWSNPLETTLPNAPEMEIFTFYGTGLLTERAYIYKLSPTAECHIPFQIDSSTNDEETCLKGGVYSVDGDGTVPVLSAGFMCAKAWHGKTKFNPSGIRTYLREYRHSPPTTLLEGRGTLSCAHVDIMRNFALIEDVIRIAAGASGEELGGNRVHSNIFKWSEKINLQL

>Gh_A08G2194

MNKPLSFYRSYFLSKTMSFLRRRNSTDSSKTRIADPNIEKDGADDDDKKKDQYKIPQRRNSGSKKWSCWDNCCWFIGFICSIWWFCLFLYNTMPDSIPQFVTMAITGPLPDPPGVKLRKEGLTANHPVVLVPGVVTGGLELWEGRHCAEGLLGKRLWGGSFGEFYKRPLCWLEHISLDNDTGFDPPGIRVRPVTGLVAADYFATGYFVWAVVIANLAHIGYEEKSLYMAAYDWRLSFQNTEIRDQSLTRIKSNIELLVATHGGKKVVVLPHSMGVQYFLHFMKWVETPAPRGGGGGPDWCAKHIKAIMNIGAPFLGSPKSIALHFSIEVRDIAALRAQAPGLLDKDVLGLQTFKHLMRMFRTWDATFSMIPKGGETIWGGLDWSPETETVNNSAKKGTDNSTRNTGGNGNICDTKGVNYGRLISFSKDWAETHSSKIERVDFKHVAKGDKLTNSSNCDIWLEYHEMGNRAIKAVADHEVYTAGSILDLLHYVAPKLMARGASHFSYGIADDLDDPKYDQYKYWSNPLETKLPNAPDMEIYSLYGVGLPTERGYIYKVAPPSDCSIPFQIDTSVDGDSEDSCLRGGVFSGDGDETVPIISAGFMCAKGWRGKTRFNPSGIRTYNREYKHAPPANLLEGRGTQSGSHVDLLGNFALIEDVLRVAAGATGEDIGGDRVYSDIFKWSERINIKL

>Gh_D06G2068

MPASFPKYVTEVITGPLPDPPGVKLRKLGLTAKHPVVFVPAAVTGGLELWEGRKCAQGLFRKRLWGGTFGDVYKRPQCWVEHMSLDNETGMDPCGIRVRPISGLVTADGLGSGYFVWTTLVSNLARIGYEEKTMFMAAYDWRLSFQNTQVRDQTLSRIKNNIELMVATRGGQKAVIIPHSAGALFFLHFMKWVEAPPPMGGGGGPYWCSKHIKAVVNIAAPFLGVPKAIPLFLSAESKDVSLVRAIAPGFLENDIFQPQILQHVMRLSHSWDSTVSMIPRGGDTIWGSLDWSPEEGYSCDQKREMKNSTRVTNPARAKSAISLTRSANFGRIVSFGKDVAEAPSNDIDRIDFKGAVKGHRAENRTCRDLWAGYHGMGFEGTKAVADYKTYTAESIVELLHFVAPKMMTRSTAHFSYGIADNMDDPKYKHYKYWSNPLETTLPIAPEMEIFSFYGTGLPTERSYVYKLSPTAECHIPFQIDSSANDEETCLKGGAYSVDGDGTVPVLSAGFMCAKAWHGKTKFNPSGIRTYVREYRHSPPTTLLEGCGTLNCAHVDIMRSFALIEDVIRIAAGASGEELGGNRVHSNIFKWSEKINLQL

>Gh_D13G2335

MASNFLRLRKPYYVESTINCSSSSDQKPVNLSFGINETRNQTKKRQAKEHHGRRSCLDSCCWVTGYLCTTWWLLLFLYHCCLPFTVGHEVPELMPAARLKREGLKGIHPVVLVPGIVTGGLELWEGKPCADGLFRKRLWGGGGFTQLLKRPLCWLEHLSLHNETGLDPPGIRVRPVPGLFGADYSAPGYFVWDVLVKNLAKIGYEGKNLHMAAYDWRLSFQNTEVRDCALSKLKSKIELMYRANGNKKVVVVPHSMGVAYFLHFLKWVETPPPMGGGGGPGWCAEHIKAIVNIGPSFLGVPKAVSNILSAEGKDVAYFRAMAPGVLDSGTLGLRALEHVMRVSRTWDSVVSLMPKGGETIWGNMDWSPEDEHVCEFSKKRYFRFSPSDNNVNNSDAKQVFRVKDPVKYGRIISFGKAASVLHSSQLPTTVLKEILHMSASRNFNSSCGEAWTEYDEMSRESIRKIGAADKAYTATTLFDLLHFVAPKMMNRAEAHFSHGIADNLEDPKYNHYKYWSNPLEMKLPDAPNMEIYCSYGVGIPTERSNVYKLSPSNKCKHIPYQIDTLVDEEDGSCLKSGVYFADGDESVPVLSAGFMCAKGWKGRTRFNPSGINTYVREYQSKPPTSGIKSTAHVDIMGNIALIEDKLRVAAGATGEEIGGDKVYSDILRMSERINLQL

>Gh_D09G0766

MSSLRRRKPINESSDSKHNEEEEDEDHDDVDADGDDDVNGKNKKTPSKIKKKRGEKPPKQPKWSCMDTCCWFIGCICIIWWLLLFLYNAMPASFPQYVTEAITGPLPDPPGVKLKKEGLEAKHPVVFVPGIVTGGLELWEGRECAEGLFRKRLWGGTFGEVYKRPLCWVEHMSLDNETGLDPCGIRVRPVSGLVAADYFAPGYFVWAVLIANLARIGYEDKTMYMAAYDWRLSFQNTEVRDQTLSRIKSNIELLVATNGGRKVVVIPHSMGVLYFLHFMKWVEAPAPMGGGGGPDWCSKHIKAVVNIGGPFLGVPKAIAGLFSAEAKDIAVARALAPGFLDNDIFQFQTLQHVMRMSRTWDSTMSMIPRGGNTIWGGLDWSPEEGNSCAKKREKKNETQIADQAGSENAVCKAKSANYGRIISFGKDVAEAPSSDIERIDFRGAIKGHSAANTTCRDVWTEYHDMGFAGIKAVAEYKTYTADSLVDLLHFVAPKMMARGTAHFSYGVADNLDDPQYKHYKYWSNPLETRLPNAPDMEIYSLYGVGLPTERAYVYKLSPHAECSIPFKIDTSADDEDTCLRDGVYSVDGDETVPVLSAGFMCAKGWRGKTRFNPSGIRTYIREYNHLPPANLLEGRGTLSGAHVDIMGNFALIEDVIRIAAGASGEELGGDQVYSKIFNWSEKINLRLVHISILLRLFIFLEVSISI

>Gh_D06G0763

MSFLRRRKGTDSSKSQNLRCNIVKEDEKNNLYESPRKESYNSKKWSCWDSCCWFIGFVCSVWWFLLLLYNTMPASIPQYVTEAIKGPLTDPPGVKLRKEGLTVNHPVVFVPGIVTGGLELWEGHQCAEGLFRKRLWGGSFGELYKRPLCWAQHMSLDNETGLDPPGIRVRPVSGLVAADYFAAGYFVWAVLIANLAQIGYEEKTMYMAAYDWRLSFQNTEVRDQTLSRIKSNIELMVATNGGKKVVVIPHSMGVLYFLHFMKWVEAPPPMGGGGGSEWCAKHIKAVMNIGGPFLGVPKSVSGLFSVEARDIAIARTFAPGFLDKDVFGLQTFQHLMRMTRTWDSTMSMIPKGGETIWGGLDWSPEGSSFNCGAKTLRNNSINNTDQIPNINLANMKNVNYGRIISFGKDVAEAHSSKIERVDLLQDSMKADMLANLTNCDIWIEYHDIGDHRIKAVADYKVYTAGSILDLLHFVSPKLMARGGAHFSYGIADNLDDPKYQHYKYWSNPLETKLPDAPDMEIYSMYGVGIPTERAYVYKVTTATDCSIPFQIDTSAEGGSEDSCLKGGVFSADGDETVPVLSAGLMAAKGWRGKTRFNPSGIPAYIREYSHAPPANLLEGRGTQSGAHVDIMGNFALIEDVIRVAAGATGKELGGDHVYSDIFKWAERINLQL

>Gh_A06G0670

MSFLRRRKGTDSSKSQNLRCNIVKEDEKNNLYESPRKESYNSKKWSCWDSCCWFIGFVCSVWWFLLLLYNTMPASIPQYVTEAIKGPLTDPPGVKLRKEGLTVNHPVVFVPGIVTGGLELWEGHQCAEGLFRKRLWGGSFGELYKRPLCWAQHMSLDNETGLDPPGIRVRPVSGLVAADYFAAGYFVWAVLIANLAQIGYEEKTMYMAAYDWRLSFQNTEVRDQTLSRIKSNIELMVATNGGKKVVVIPHSMGVLYFLHFMKWVEAPPPMGGGGGSEWCAKHIKAVMNIGGPFLGVPKSVSGLFSVEARDIAIARTFAPGFLDKDVFGLQTFQHLMRMTRTWDSTMSMIPKGGETIWGGLDWSPEGSSFNCGAKTLRNNSINNTDQIPNINLANMKNVNYGRIISFGKDVAEAHSSKIERVDLLQDSMKADMLANLTNCDIWIEYHDIGDHRIKAAADYKVYTAGSILDLLHFVSPKLMARGGAHFSYGIADNLDDPKYQHYKYWSNPLETKLPDAPDMEIYSMYGVGIPTERAYVYKVTTATDCSIPFQIDTSVEGGSEDSCLKGGVFSADGDETVPVLSAGLMAAKGWRGKTRFNPSGIPTYIREYSHAPPANLLEGRGTQSGAHVDIMGNFALIEDIIRVAAGATGKELGGDHVYSDIFKWAERINLQL

>Gh_A07G0967

MASVLRFRKLCYVESAVKCTSVGYESFEIDEKLDKKEEDVLSANNFALEINKKRKLPKRQPKEWRCLDSCCWIIGYLCTTWWLLLFFYHCFPVTLLRVPELPGPGVRLKREGLTALHPVVLVPGIVTGGLELWEGRPCADGLFRKRLWGGGSFTQIFKRPLCLLEHLSLHYETGLDPQGIRVRAVPGLVGADYFAPGYFVWAVLIENLAKIGYEGKNLHMAAYDWRLSFQNTEIRDHALTRLKSKIELMYISNGYKKVVAVPHSMGVIYFLHFLKWVETPPPMGGGGGPGWCAKHIKAVMNIGPAFLGVPKAVSNLFSAEGKDVSYIRAMAPGVFDSEILGLQTFERVMRMARTWDSIVSLLPKGGEVIWGNMDRSPEEGHVCDFSKKSHSKTSLTTNNVNNSDVKRGFLVKDLANYGRIISFGKPASILHSSKLPTSDSKVFSRTSTSENFNNFSCGEAWTEYDEMSRQSIQNVAADKAYTTTTLLDLLRFVAPKMMRRAEAHFSHGIAENLDDPKYNHYKYWSNPLETKLSDAPDMEIYCMYGVGIPTERSYVYKLSPNSRCKSIPYQIDNSVHGEDGSCLKGGVYFADGDESVPVLSAGFMCAKGWRGRTRFNPSGIATYIREYRHKPPTSLMEGRGIESGSHVDIMGNFALIEDIMRVASGATGDEIGGDQIHSDIIKMSERINLGL

>Gh_D08G2559

MSKPLSFYRSYFLSKTMSFLRRRNGTDSSKTRVADPNIKKDGADDDDKKKDQYKIPQRRNYGSKKWSCWDNCCWFIGFICSIWWFCLFLYNTMPDSIPQFVTMAITGPLPDPPGVKLRKEGLTANHPVVLVPGVVTGGLELWEGRHCAEGLLGKRLWGGSFGEFYKRPLCWLEHISLDNDTGFDPPGIRVRPVTGLVAADYFATGYFVWAVVIANLAHIGYEEKSLYMAAYDWRLSFQNTEIRDQSLTRIKSNIELLVATHGGKKVVVLPHSMGVQYFLHFMKWVETPAPRGGGGGPDWCAKHIKAIMNIGAPFLGSPKSIALHFSIEVRDIAALRAQAPGLLDKDVLGLQTFKHLMRMFRTWDATFSMIPKGGETIWGGLDWSPETETVNNSAKKGTDNSTRNTGGNGNICNTKGVNYGRLISFSKDWAETHSSKIERVDFKHVAKGDKLTNSSNCDIWLEYHEMGNRAIKAVADHKVYTAGSILDLLHYVAPKLMARGASHFSYGIADDLDDPKYDQYKYWSNPLETKLPNAPDMEIYSLYGVGLPTERGYIYKVAPPSDCSIPFQIDTSVDGDSEDSCLKGGVFSGDGDETVPIISAGFMCAKGWRGKTRFNPSGIRTYNREYKHAPPANLLEGRGTQSGSHVDLLGNFALIEDVLRVAAGATGEDIGGDRVYSDISKWSERINIKL

>Gh_A13G1940

MASNFLRLRKPYYVESTINCSSSSDQKLVNLSFGIDETRNQTKKRQAKEHHGRRSCLDSCCWVTGYLCTTWWLLLFLYHCCLPFTVVHEVPQLMPAARLKREGLTGIHPVVLVPGIVTGGLELWEGKPCADGLFRKRLWGGGGFTQLLKRPLCWLEHLSLHNETGLDPPGIRVRPVPGLFGADYSAPGYFVWDVLVKNLAKIGYEGKNLHMAAYDWRLSFQNTEVRDCALSRLKSKIELMYRANGNKKVVVVPHSMGVAYFLHFLKWVETPPPMGGGGGLGWCAEHIKAIVNIGPSFLGVPKAVSNILSAEGKDVAYFRAMAPGVLDSGTLGLRALEHVMRVSRTWDSVVSLMPKGGETIWGNMDWSPEDEHVCDFSKKRYFRLSPSDNNVNKSNAKQVFRVKDPVKYGRIISFGKAASVLHSSQLPTAVLKEILHMSASRNFTSSCGEAWTEYDEMSRESIQKIGAADKAYTATTLFDLLRFVAPKMMNRTEAHFSHGIADNLDDPKYNHYKFWSNPLEMKLPDAPNMEIYCSYGVGIPTERSYVYKLSPSNKCKRIPYQIDTSVDGEDRSCLKSGVYFADGDESVPVLSAGFMCAKGWKGRTRFNPSGINTYVREYQSKPLTSGIKSTAHVDIMGNIALIEDILRVAAGATGEEIGGNKIYSDILRMSERINLRL

>Gh_A09G0767

MSSLRRRKPINESSDSKHKAEEEDEDHDDVDADGDDDVNGKNKKTPSKIKKKRGEKPPKQPKWSCMDTCCWFIGCICITWWLLLFLYNAMPASFPQYVTEAITGPLPDPPGVKLKKEGLEAKHPVVFVPGIVTGGLELWEGRECAEGLFRKRLWGGTFGEVYKRPLCWVEHMSLDNETGLDPCGIRVRPVSGLVAADYFAPGYFVWAVLIANLARIGYEDKTMYMAAYDWRLSFQNTEVRDQTLSRIKSNIELLVATNGGRKVVVIPHSMGVLYFLHFMKWVEAPAPMGGGGGPDWCSKHIKAVVNIGGPFLGVPKAIAGLFSAEAKDIAVARALAPGFLDNDIFQFQTLQHVMRMSRTWDSTMSMIPRGGNTIWGGLDWSPEEGNSCAKKREKKNETQIADQAGSENAVCKAKSANYGRIISFGKDVAEAPSSDIERIDFRGAIKGHSAANTTCRDVWTEYHDMGFAGIKAVAEYKTYTADSLVDLLHFVAPKMMARGTAHFSYGVADNLDDPKYKHYKYWSNPLETRLPNAPDMEIYSLYGVGLPTERAYVYKLSPHAECSIPFKIDTSADDEDTCLRDGVYSVDGDETVPVLSAGFMCAKGWRGKTRFNPSGIRTYIREYNHLPPANLLEGRGTLSGAHVDIMGNFALIEDVIRIAAGASGEELGGDQVYSKIFNWSENINLQLVHISILLRLFIFLEVSISI

>Gh_D07G1046

MASVLRFRKLCYVESAVKCTSVGYESFEIDEKLDEKEEDVLSANNFALEINKKRKLPKRQPKEWRCLDSCCWIIGYLCTTWWLLLFCYHCLPVTLLRVPELPGPGVRLKREGLTALHPVVLVPGIVTGGLELWEGRPCADGLFRKRLWGGGSFTQIFKRPLCLLEHLSLHYETGLDPQGIRVRAVPGLVGADYFAPGYFVWAVLIENLAKIGYEGKNLHMAAYDWRLSFQNTEIRDHALTRLKSKIELMYISNGYKKVVAVPHSMGVIYFLHFLKWVETPPPMGGGGGPGWCAKHIKAVMNIGPAFLGVPKAVSNLFSAEGKDVSYIRAMAPGVFDSEILGLQTFERVMRMARTWDSIVSLLPKGGEVIWGNMDRSPEEGHVCDFSKKSHSKTSLTTNNVNNSDVKRGFLVKDLANYGRIISFGKPASVLHSSKLPTADSKEFSRTSTSENFNNFSCGEAWTEYDEMSRQSIQNVAADKAYTTTTLLDLLRFVAPKMMRRAEAHFSHGIAENLDDPKYNHYKYWSNPLETKLSDAPDMEIYCMYGVGIPTERSYVYKLSPNSRCKSIPYQIDNSVHGEDGSCLKGGVYFADGDESVPVLSAGFMCAKGWRGRTRFNPSGIATYIREYRHKPPTSLMEGRGIESGSHVDIMGNFALIEDIMRVAAGATGDEIGGDQIHSEIIKMSERINLGL

>Cotton_D_gene_10010901

MSSLRRRKPINESSDSKHNEEEEDEDHDDVDADGDDDVNGKNKKTPSKIKKKRGEKPPKQPKWSCMDTCCWFIGCICITWWLLLFLYNAMPASFPQYVTEAITGPLPDPPGVKLKKEGLEAKHPVVFVPGIVTGGLELWEGRECAEGLFRKRLWGGTFGEVYKRPLCWVEHMSLDNETGLDPCGIRVRPVSGLVAADYFAPGYFVWAVLIANLARIGYEDKTMYMAAYDWRLSFQNTEVRDQTLSRIKSNIELLVATNGGRKVVVIPHSMGVLYFLHFMKWVEAPAPMGGGGGPDWCSKHIKAVVNIGGPFLGVPKAIAGLFSAEAKDIAVARALAPGFLDNDIFQFQTLQHVMRMSRTWDSTMSMIPRGGNTIWGGLDWSPEEGNSCAKKGEKKNETQIADQAGSENAVCKAKSANYGRIISFGKDVAEAPSSDIERIDFRGAIKGHSAANTTCRDVWTEYHDMGFAGIKAVAEYKTYTADSLVDLLHFVAPKMMARGTAHFSYGVADNLDDPQYKHYKYWSNPLETRLPNAPDMEIYSLYGVGLPTERAYVYKLSPHAECSIPFKIDTSADDEDTCLRDGVYSVDGDETVPVLSAGFMCAKGWRGKTRFNPSGIRTYIREYNHLPPANLLEGRGTLSGAHVDIMGNFALIEDVIRIAAGASGEELGGDQVYSKIFNWSEKINLRLVHISILLQLFIFLEVSISI

>Cotton_D_gene_10039077

MSFLRRRKGTDSSKSQNLRCSIVKEDEKNNLYESPRKESYNSKKWSCWDSCCWFIGFVCSVWWFLLLLYNTMPASIPQYVTEAITGPLTDPPGVKLRKEGLTVNHPVVFVPGIVTGGLELWEGHQCAEGLFRKRLWGGSFGELYKRLEVIREASIKLTDFYMKLIPLCWAQHMSLDNETGLDPPGIRVRPVSGLVAADYFAAGYFVWAVLIANLAQIGYEEKTMYMAAYDWRLSFQNTEVRDQTLSRIKSNIELMVATNGGKKVVVIPHSMGVLYFLHFMKWVEAPPPTGGGGGSEWCAKHIKAVMNIGGPFLGVPKSVSGLFSVEARDIAIARTFAPGFLDKDVFGLQTFQHLMRMTRTWDSTMSMIPKGGETIWGGLDWSPEGSSFNCGAKTLRNNSINNTDQIPNINLANMKNVNYGRIISFGKDVAEAHSSKIERVDLLDSMKADMLANLTNCDIWIEYHDIGDHRIKAVADYKVYTAGSILDLLHFVSPKLMARGGAHFSYGIADNLDDPNYQHYKYWSNPLETKLPDAPDMEIYSMYGVGIPTERAYVYKVTTATDCSIPFQIDTSAEGGSEDSCLKGGVFSADGDETVPVLSAGLMAAKGWRGKTRFNPSGIPTYIREYSHAPPANLLEGRGTQSGAHVDIMGNFALIEDVIRVAAGATGKELGGDHVYSDIFKWAERINLQL

>Cotton_D_gene_10010224

MPDSIPQFVTMAITGPLPDPPGVKLRKEGLMANHPVVLVPGVVTGGLELWEGRHCAEGLLGKRLWGGSFGEFYKRPLCWLEHISLDNDTGFDPPGIRVRPVTGLVAADYFATGYFVWAVVIANLAHIGYEEKSLYMAAYDWRLSFQNTEIRDQSLTRIKSNIELLVATHGGKKVVVLPHSMGVQYFLHFMKWVETPAPRGGGGGPDWCAKHIKAIMNIGAPFLGSPKSIALHFSIEVRDIAALRAQAPGLLDKDVLGLQTFKHLMRMFRTWDATFSMIPKGGETIWGGLDWSPETETVNNSAKKGTDNSTRNTGGNGNICNTKGVNYGRLISFSKDWAETHSSKIERVDFKHVAKGDKLTNSSNCDIWLEYHEMGNRAIKAVADHKVYTAGSILDLLHYVAPKLMARGASHFSYGIADDLDDPKYDEYKYWSNPLETKLPNAPDMEIYSLYGVGLPTERGYIYKVAPPSDCSIPFQIDTSVDGDSEDSCLKGGVFSGDGDETVPIISAGFMCAKGWRGKTRFNPSGIRTYNREYKHAPPANLLEGRGTQSGSHVDLLGNFALIEDVLRVAAGATGEDIGGDRVYSDIFKWSERINIKL

>Cotton_D_gene_10023304

MSLLRRRKHPFNESFRKPEKEHENGDFSYESKRKKIPYEHEEERPLSKWSCLDTWCWLIGCVCVTWWFLLFLYNAMPASFPKYVTEVITGPLLDPPGVKLRKLGLTAKHPVVFVPAAVTGGLELWEGRKCAQGLFRKRLWGGTFGDVYKRPQCWVEHMSLDNETGMDPCGIRVRPISGLVTADGLGSGCFVWTTLVSNLARIGYEEKTMFMAAYDWRLSFQNTQVRDQTLSRIKKNIELMVATSGGQKAVIIPHSAGALFFLHFMKWVEAPPPMGGGGGPYWCSKYIKAVVNIAAPFLGVPKAIPLFLSAESKDISLVRAIAPGFLENDIFQPQILQHVMRLSHSWDSTVSMIPRGGDTIWGSLDWSPEEGYSCDQKREMKNSTRVTNPARAKSAISLTRSANFGRIVSFGKDVAEAPSNDIDRIDFKGAVKGHRAENRTCRDLWAEYHGMGFEGTKAVADYKTYTAESIVELLHFVAPKMMARSTAHFSHGIADNMDDPKLPIAPEMEIFSFYGTGLPTERSYVYKLSPTAECHIPFQIDSSANDEETCLKGGAYSVDGDGTVPVLSAGFMCAKAWHGKTKFNPSGIRTYVREYRHSPPTTLLEGCGTLNCAHVDIMRNFAVIEDVIRIAAGASGEELGGNRVHSNIFKWSEKINLQL

>Cotton_D_gene_10012986

MASVLRFRKLCYVESAVKCTSVGYESFEIDEKLDEKEEDVLSANHFALEINKKRKQPKRQPKEWRCLDSCCWIIGYLCTTWWLLLFCYHCLPVTLLRVPELPGPGVRLKREGLTALHPVVLVPGIVTGGLELWEGRPCADGLFRKRLWGGGSFTQIFKRPLCLLEHLSLHYETGLDPQGIRVRAVPGLVGADYFAPGYFVWAVLIENLAKIGYEGKNLHMAAYDWRLSFQNTEIRDHALTRLKSKIELMYISNGYKKVVAVPHSMGVIYFLHFLKWVETPPPMGGGGGPGWCAKHIKAVMNIGPAFLGVPKAVSNLFSAEGKDVSYIRAMAPGVFDSEILGLQTFERVMRMARTWDSIVSLLPKGGEVIWGNMDRSPEEGHVCDFSKKSHSKTSLTTNNINNSDVKRGFLVKDLANYGRIISFGKPASVLHSSKLPTADSKEFSRTSTSENFNNFSCGEAWTEYDEMSRQSIQNVAADKAYTTTTLLDLLRFVAPKMMRRAEAHFSHGIAENLDDPKYNHYKYWSNPLETKLSDAPDMEIYCMYGVGIPTERSYVYKLSPNSRCKSIPYQIDNSVHGEDGSCLKGGVYFADGDESVPVLSAGFMCAKGWRGRTRFNPSGIATYIREYRHKPPTSLMEGRGIESGSHVDIMGNFALIEDIMRVAAGATGDEIGGDQIHSDIIKMSERINLGL

>Cotton_D_gene_10025228

MASNFLRLRKPYYVESTINCSSSSDQKLVNLSFGINETRNQTKKRQAKEHHGRRSCLDSCCWVTGYLCTTWWLLLFLYHCCLPFTVGHEVPELMPAARLKREGLKGIHPVVLVPGIVTGGLELWEGKPCADGLFRKRLWGGGGFTQLLKRPLCWLEHLSLHNETGLDPPGIRVRPVPGLFGADYSAPGYFVWDVLVKNLAKIGYEGKNLHMAAYDWRLSFQNTEVRDRALSKLKSKIELMYRANGNKKVVVVPHSMGVAYFLHFLKWVETPPPMGGGGGPGWCAEHIKAIVNIGPSFLGVPKAVSNILSAEGKDVAYFRAMAPGVLDSGTLGLRALEHVMRVSRTWDSVVSLMPKGGETIWGNMDWSPEDEHVCDFSKKRYFRFSPSDNNVNNSDAKQVFRVKDPVKYGRIISFGKAASVLHSSQLPTTVLKEILHMSASRNFNSSCWEAWTEYDEMSRESIRKIGAADKAYTATTLFDLLRFVAPKMMNRAEAHFSHGIADNLEDPKYNHYKYWSNPLEMKLPDAPNMEIYCSYGVGIPTERSYVYKLSPSNKCKHIPYQIDTSVDGEDGSCLKSGVYFADGDESVPVLSAGFMCAKGWKGRTRFNPSGINTYVREYQSKPPTSGIKSTAHVDIMGNIALIEDILRVAAGATGEEIGGDKIYSDILRMSERINLRL

>Cotton_A_28721

MPASFPQYVTEAITGPLPDPPGVKLKKEGLEAKHPVVFVPGIVTGGLELWEGRECAEGLFRKRLWGGTFGEVYKRPLCWVEHMSLDNETGLDPCGIRVRPVSGLVAADYFAPGYFVWAVLIANLARIGYEDKTMYMAAYDWRLSFQNTEVRDQTLSRIKSNIELLVATNGGRKVVVIPHSMGVLYFLHFMKWVEAPAPMGGGGGPDWCSKHIKAVVNIGGPFLGVPKAIAGLFSAEAKDIAVARALAPGFLDNDIFQFQTLQHVMRMSRTWDSTMSMIPRGGNTIWGGLDWSPEEGNSCAKKREKKNETQIADQAGSENAVCKAKSANYGRIISFGKDVAEAPSSDIERIDFRGAIKGHSAANTTCRDVWTEYHDMGFAGIKAVAEYKTYTADSLVDLLHFVAPKMMARGTAHFSYGVADNLDDPKYKHYKYWSNPLETRLPNAPDMEIYSLYGVGLPTERAYVYKLSPHAECSIPFKIDTSADDEDTCLRDGVYSVDGDETVPVLSAGFMCAKGWRGKTRFNPSGIRTYIREYNHLPPANLLEGRGTLSGAHVDIMGNFALIEDVIRIAAGASGEELGGDQVYSKIFNWSENINLQLDEEIPVHSSS

>Cotton_A_06686

MSFSRRRKGTDSSKSQNLRCNIVKEDEKNNLYESPRKESYNSKKWSCWDSCCWFIGFVCSVWWFLLLLYNTMPASIPQYVTEAITGPLTDPPGVKLRKEGLTVNHPVVFVPGIVTGGLELWEGHQCAEGLFRKRLWGGSFGELYKRLEVIREASIKLTDHEIDVWLCFAKTKTFSLLQIPLCWAQHMSLDNETGLDPPGIRVRPVSGLVAADYFAAGYFVWAVLIANLAQIGYEEKTMYMAAYDWRLSFQNTEVRDQTLSRIKSNIELMVATNGGKKVVVIPHSMGVLYFLHFMKWVEAPPPMGGGGGSEWCAKHIKAVMNIGGPFLGVPKSVSGLFSVEARDIAIARTFAPGFLDKDVFGLQTFQHLMRMTRTWDSTMSMIPKGGETIWGGLDWSPEGSSFNCGAKTLRNNSINNTDQIPNINLANMKNVNYGRIISFGKDVAEAHSSKIKRVDLLQDSMKADMLANLTNCDIWIEYHDIGDHRIKAVADYKVYTAGSILDLLHFVSPKLMARGGAHFSYGIADNLDDPKYQHYKYWSNPLETKLPDAPDMEIYSMYGVGIPTERAYVYKVTTATDCSIPFQIDTSVEGGSEDSCLKGGVFSADGDETVPVLSAGLMAAKGWRGKTRFNPSGIPTYIREYSHAPPANLLEGRGTQSGAHVDIMGNFALIEDIIRVAAGATGKELGGDHVYSDIFKWAERINLQL

>Cotton_A_05247

MSLLRRRKHPFNESFRKPEKEHENGDFSYESKRKKIPYEHEEERPLSKWSCFDTWCWLIGCVCVTWWFILFLYNAMPASFPKYVTEVITGPLPDPPGVKLRKLGLTAKHPVVFVPAAVTGGLELWEGRKCAQGLFRKRLWGGTYGDVYKRPQCWVEHMSLDNETGMDPCGIRVRPISGLVTADGFGSGCFVWTTLVSNLARIGYEEKTMFMAAYDWRLSFQNTQVRDQTLSRIKNNIELMVATSGGQKVVIIPHSTGALFFLHFMKWVEAPPPMGGGGGPYWCSKHIKAVVNIAAPFLGVPKAIPLFLSAESKDISLVRAIAPGFFENDIFQPQILQHVMRLSHSWDSTVSMIPRGGDTIWGGLDWSPEEGYSCDQKREMKNSTLVTNPARAKSAISLTRSANFGRIVSFGKDVAEAPSNDIDRIDFKGAVKGHRAENRTCRDLWTEYNGMGFEGTKAVADYKTYTAESIVELLHFVAPKMMARSTAHFSYGIADNMDDPKYKHYKYWSNPLETTLPNAPEMEIFSFYGTGLLTERAYIYKLSPTAECHIPFQIDSSTNDEETCLKGGVYSVDGDGTVPVLSAGFMCAKAWHGKTKFNPSGIRTYLREYRHSPPTTLLEGRGTLSCAHVDIMRNFALIEDVIRIAAGASGEELGGNRVHSNIFKWSEKINLQL

>Cotton_A_11856

MSFLRRRNSTDSSKTRIADPNIEKDGADDGDKKKDQYKIPQRRNSGSKKWSCWDNCCWFIGFICSIWWFCLFLYNTMPDSIPQFVTMAITGPLPDPPGVKLRKEGLTANHPVVLVPGVVTGGLELWEGRHCAEGLLGKRLWGGSFGEFYKRCIVRSACYMLKSPHFVTGLESRNLLLPLCWLEHISLDNDTGFDPPGIRVRPVTGLVAADYFATGYFVWAVVIANLAHIGYEEKSLYMAAYDWRLSFQNTEIRDQSLTRIKSNIELLVATHGGKKVVVLPHSMGVQYFLHFMKWVETPAPRGGGGGPDWSAKHIKAIMNIGAPFLGSPKSIALHFSIEVRDIAALRAQAPGLLDKDVLGLQTFKHLMRMFRTWDATFSMIPKGGETIWGGLDWSPETETVNNSAKKGTDNSTRNTGGNGNICDTKGVNYGRLISFNKDWAETHSSKIERVDFKQHVAKGDKLTNSSNCDIWLEYHEMGNRAIKAVADHKVYTAGSILDLLHYVAPKLMARGASHFSYGIADDLDDPKYDQYKYWSNPLETKLPNAPDMEIYSLYGVGLPTERGYIYKVAPPSDCSIPFQIDTSVDGDSEDSCLRGGVFSGDGDETVPIISAGFMCAKGWRGKTRFNPSGIRTYNREYKHAPPANLLEGRGTQSGSHVDLLGNFALIEDVLRVAAGATGEDIGGDRVYSDIFKWSERINIKL

>Cotton_A_09547

MASVLRFRKLCYVESAVKCTSVGYESFEIDEKLDKKEEDVLSANNFALEINKKRKQPKRQPKEWRCLDSCCWIIGYLCTTWWLLLFFYHCFPVTLLRVPELPGPGVRLKLEGLTALHPVVLVPGIVTGGLELWEGRPCADGLFRKRLWGGGSFTQIFKRPLCLLEHLSLHYETGLDPQGIRVRAVPGLVGADYFAPGYFVWAVLIENLAKIGYEGKNLHMAAYDWRLSFQNTEIRDHALTRLKSKIELMYISNGYKKVVAVPHSMGVIYFLHFLKWVETPPPMGGGGGPGWCAKHIKAVMNIGPAFLGVPKAVSNLFSAEGKDVSYIRAMAPGVFNSEILGLQTFERVMRMARTWDSIVSLLPKGGEVIWGNMDRSPEEGHVCDFSKKSHSKTSLTTNNVNNSDLKRGFLVKDLANYGRIISFGKPASILHSSKLPTSDSKVFSRTSTSENFNNFSCGEAWTEYDEMSRQSIQNVAADKAYTTTTLLDLLRFVAPKMMRRAEAHFSHGIAENLDDPKYNHYKYWSNPLETKLSDAPDMEIYCMYGVGIPTERSYVYKLSPNSRCKSIPYQIDNSVHGEDGSCLKGGVYFADGDESVPVLSAGFMCAKGWRGRTRFNPSGIATYIREYRHKPPTSLMEGRGIESGSHVDIMGNFALIEDIMRVASGATGDEIGGDQIHSDIIKMSERINLGL

>Cotton_A_20000

MLMFRPLCWLEHLSLHNETGLDPPGIRVRPVPGLFGADYSAPGYFVWDVLVKNLAKIGYEGKNLHMAAYDWRLSFQNTEVRDRALSRLKSKIELMYRANGNKKVVVVPHSMGVAYFLHFLKWVETPPPMGGGGGLGWCAEHIKAIVNIGPSFLGVPKAVSNILSAEGKDVAYFRAMVPGVLDSGTLGLRALEHVMRVSRTWDSVVSLMPKGGETIWGNMDWSPEEEHVCDFSKKRYFRLSPSDNNVNKSNAKQVFRVKDPVKYGRIISFGKAASVLHSSQLPTAVLKEILHMSASRNFNSSCGEAWTEYDEMSRESIQKIGAADKAYTATTLFDLLRFVAPKMMNRTEAHFSHGIADNLDDPKYNHYKFWSNPLEMKLPDAPNMEIYCSYGVGIPTERSYVYKLSPSNKCKRIPYQIDTSVDGEDRSCLKSGVYFADGDESVPVLSAGFMCAKGWKGRTRFNPSGINTYVREYQSKPLTSGIKSMAHVDIMGNIALIEDILRVAAGATGEEIGGNKIYSDILRMSERINLRL

>Gbscaffold10824.9.0

MSSLRRRKPINESSDSKHKAEEEDEDHDDVDADGDDDVNGKNKKTPSKIKKKRGEKPPKQPKWSCMDTCCWFIGCICITWWLLLFLYNAMPASFPQYVTEAITGPLPDPPGVKLKKEGLEAKHPVVFVPGIVTGGLELWEGRECAEGLFRKRLWGGTFGEVYKRPLCWVEHMSLDNETGLDPCGIRVRPVSGLVAADYFAPGYFVWAVLIANLARIGYEDKTMYMAAYDWRLSFQNTEVRDQTLSRIKSNIELLVATNGGRKVVVIPHSMGVLYFLHFMKWVEAPAPMGGGGGPDWCSKHIKAVVNIGGPFLGVPKAIAGLFSAEAKDIAVARALAPGFLDNDIFQFQTLQHVMRMSRTWDSTMSMIPRGGNTIWGGLDLSPEEGNSCAKKREKKNETQIADQAGSENAVCKAKSANYGRIISFGKDVAEAPSSDIERIDFRGAIKGHSAANTTCRDVWTEYHDMGFAGIKAVAEYKTYTADSLVDLLHFVAPKMMARGTAHFSYGVADNLDDPQYKHYKYWSNPLETRLPNAPDMEIYSLYGVGLPTERAYVYKLSPHAECSIPFKIDTSADDEDTCLRDGVYSVDGDETVPVLSAGFMCAKGWRGKTRFNPSGIRTYIREYNHLPPANLLEGRGTLSGAHVDIMGNFALIEDVIRIAAGASGEELGGDQVYSKIFNWSEKINLRLDEEIPVHSSR

>Gbscaffold1227.2.0

MFGTPEIRPLCWVEHMSLDNETGLDPCGIRVRPVSGLVAADYFAPGYFVWAVLIANLARIGYEDKTMYMAAYDWRLSFQNTEVRDQTLSRIKSNIELLVATNGGRKVVVIPHSMGVLYFLHFMKWVEAPAPMGGGGGPDWCSKHIKAVVNIGGPFLGVPKAIAGLFSAEAKDIAVARALAPGFLDNDIFQFQTLQHVMRMSRTWDSTMSMIPRGGNTIWGGLDWSPEEGNSCAKKREKKNETQIADQAGSENAVCKAKSANYGRIISFGKDVAEAPSSDIERIDFRGAIKGHSAANTTCRDVWTEYHDMGFAGIKAVAEYKTYTADSLVDLLHFVAPKMMARGTAHFSYGVADNLDDPKYKHYKYWSNPLETRLPNAPDMEIYSLYGVGLPTERAYVYKLSPHAECSIPFKIDTSADDEDTCLRDGVYSVDGDETVPVLSAGFMCAKGWRGKTRFNPSGIRTYIREYNHLPPANLLEGRGTLSGAHVDIMGNFALIEDVIRIAAGASGEELGGDQVYSKIFNWSENINLQLDEEIPVHSSS

>Gbscaffold14436.10.0

MASNFLRLRKPYYVESTINCSSSSDQKPVNLSFGINETRNQTKKRQAKEHHGRRSCLDSCCWVTGYLCTTWWLLLFLYHCCLPFTVGHEVPELMPAARLKREGLKGIHPVVLVPGIVTGGLELWEGKPCADGLFRKRLWGGGGFTQLLKRYLEPLPKHKYFVWPSQNATGLDPPGIRVLPVPGLFGADYSAPGYFVWDVLVKNLAKIGYEGKNLHMAAYDWNSLSRIQRYKWLFSMCRFLVPVRDCALSKLKSKIELMYRANGNKKVVVVPHSMGVAYFLHFLKWVETPPPMGGGGGPGWCAEHIKAIVNIGPSFLGVPKAVSNILSAEGKDVAYFRAMAPGVLDSGTLGLRALEHVMRVSRTWDSVVSLMPKGGETIWGNMDWSPEDEHVCEFSKKRYFRFSPSDNNVNNSDAKQVFRVKDPVKYGRIISFGKAASVLHSSQLPTTVLKEILHMSASRNFNSSCGEAWTEYDEMSRESIRKIGAADKAYTATTLFDLLRFMAPKMMNRAEAHFSHGIADNLEDPKYNHYKYWSNPLEMKLPDAPNMEIYCSYGVGIPTERSNVYKLSPSNKCKHIPYQIDTLVDEEDGSCLKSGVYFADGDESVPVLSAGFMCAKGWKGRTRFNPSGINTYVREYQSKPPTSGIKSTAHVDIMGNIALIEDILRVAAGATGEEIGGDKIYSDILRMSERINLQL

>Gbscaffold14656.14.0

MSLLRRRKHPFNESFRKPEKEHENGDFSYESKRKKIPYEHEEERPLSKWSCLDTWCWLIGCVCVTWWFLLFLYNAMPASFPKYVTEVITGPLPDPPGVKLRKLGLTAKHPVVFVPAAVTGGLELWEGRKCAQGLFRKRLWGGTFGDVYKRPQCWVEHMSLDNETGMDPCGIRVRPISGLVTADGLGSGYFVWTTLVSNLARIGYEEKTMFMAAYDWRLSFQNTQVRDQTLSRIKNNIELMVATRGGQKAVIIPHSAGALFFLHFMKWVEAPPPMGGGGGPYWCSKHIKAVVNIAAPFLGVPKAIPLFLSAESKDISLVRAIAPGFLENDIFQPQILQHVMRLSHSWDSTVSMIPRGGDTIWGSLDWSPEEGYSCDQKREMKNSTRVTNPARAKSAISLTRSANFGRIVSFGKDVAEAPSNDIDRIDFKGAVKGHRAENRTCRDLWTEYHGMGFEGIKAVADYKTYTAESIVELLHFVAPKMMARSTAHFSHGIADNMDDPKYKHYKYWSNPLETTLPIAPEMEIFSFYGTGLPTERSYVYKLSPTAECHIPFQIDSSANDEETCLKGGAYSVDGDGTVPVLSAGFMCAKAWHGKTKFNPSGIRTYVRDQTLSRIKNNIELMVATRGGQKAVIIPHSAGALFFLHFMKWVEAPPPMGGGGGPYWCSKHIKAVVNIAAPFLGVPKAIPLFLSAESKDISLVRAIAPGFLENDIFQPQILQHVMRLSHSWDSTVSMIPRGGDTIWGSLDWSPEEGYSCDQKREMKNSTRVTNPARAKSAISLTRSANFGRIVSFGKDVAEAPSNDIDRIDFKGAVKGHRAENRTCRDLWAEYHGMGFEGTKAVADYKTYTAESIVELLHFVAPKMMARSTAHFSHGIADNMDDPKYKHYKYWSNPLETTLPIAPEMEIFSFYGTGLPTERSYVYKLSPTAECHIPFQIDSSANDEETCLKGGAYSVDGDGTVPVLSAGFMCAKAWHGKTKFNPSGIRTYVREYRHSPPTTLLEGCGTLNCAHVDIMRSFALIEDVIIAAGASGEELGGNRVHSNIFKWRL

>Gbscaffold19738.1.3

MSLDNETGLDPPGIRVRPVSGLVAADYFAAGYFVWAVLIANLAQIGYEEKTMYMAAYDWRLSFQNTEVRDQTLSRIKSNIELMVATNGGKKVVVIPHSMGVLYFLHFMKWVEAPPPMGGGGGSEWCAKHIKAVMNIGGPFLGVPKSVSGLFSVEARDIAIARTFAPGFLDKDVFGLQTFQHLMRMTRTWDSTMSMIPKGGETIWGGLDWSPEGSSFNCGAKTLRNNSINNTDQIPNINLANMKNVNYGRIISFGKDVAEAHSSKIERVDLLDSMKADMLANLTNCDIWIEYHDIGDHRIKAVADYKVYTAGSILDLLHFVSPKLMARGGAHFSYGIADNLDDPKYQHYKYWSNPLETKLPDAPDMEIYSI

>Gbscaffold21128.50.0

MNKPLSFYRSYFLSKTMSFLRRRNSTDSSKTRIADPNIEKDGADDDDKKKDQYKIPQRRNSGSKKWSCWDNCCWFIGFICSIWWFCLFLYNTMPDSVPQFVTMAITGPLPDPPGVKLRKEGLTANHPVVLVPGVVTGGLELWEGRHCAEGLLGKRLWGGSFGEFYKRPLCWLEHISLDNDTGFDPPGIRVRPVTGLVAADYFATGYFVWAVVIANLAHIGYEEKSLYMAAYDWRLSFQNTEIRDQSLTRIKSNIELLVATHGGKKVVVLPHSMGVQYFLHFMKWVETPAPRGGGGGPDWCAKHIKAIMNIGAPFLGSPKSIALHFSIEVRDIAALRAQAPGLLDKDVLGLQTFKHLMRMFRTWDATFSMIPKGGETIWGGLDWSPETETVNNSAKKGTDNSTRNTGGNGNICDTKGVNYGRLISFSKDWAETHSSKIERVDFKHVAKGDKLTNSSNCDIWLEYHEMGNRAIKAVADHKVYTAGSILDLLHYVAPKLMARGASHFSYGIADDLDDPKYDQYKYWSNPLETKLPNAPDMEIYSLYGVGLPTERGYIYKVAPPSDCSIPFQIDTSVDGDSEDSCLRGGVFSGDGDETVPIISTGFMCAKGWRGKTRFNPSGIRTYNREYKHAPPANLLEGRGTQSGSHVDLLGNFALIEDVLRVAAGATGEDIGGDRVYSDIFKWSERINIKL

>Gbscaffold24182.2.0

MSLLRRRKHPFNESFRKPEKEHENGDFSYESKRKKIAYEHEEERPLSKWSCFDTWCWLIGCVCVTWWFILFLYNAMPASFPKYVTEVITGPLPDPPGVKLRKLGLTAKHPVVFVPAAVTGGLELWEGRKCAQGLFRKRLWGGTYGDVYKRPQCWVEHMSLDNETGMDPCGIRVRPISGLVTADGFGSGCFVWTTLVSNLARIGYEEKTMFMAAYDWRLSFQNTQVRDQTLSRIKNNIELMVATSGGQKVVIIPHSTGALFFLHFMKWVEAPPPMGGGGGPYWCSKHIKAVVNIAAPFLGVPKAIPLFLSAESKDISLVRAIAPGFFENDIFQPQILQHVMRLSHSWDSTVSMIPRGGDTIWGGLDWSPEEGYSCDQKREMKNSTLVTNPARAQSAISLTRSANFGRIVSFGKDVAEAPSNDIDRIDFKGAVKGHRAENRTCRDLWTEYHGMGFEGIKAVADYKTYTAESIVELLHFVAPKMMTRSTAHFSYGIADNMDDPKYKHYKYWSNPLETTLPNAPEMEIFSFYGTGLLTERAYIYKLSPTAECHIPFQIDSSTNDEETCLKGGVYSVDGDGTVPVLSAGFMCAKAWHGKTKFNPSGIRTYLREYRHSPPTTLLEGRGTLSCAHVDIMRSFALIEDVIRIAAGASGEELGGNRVHSNIFKWSEKINLQL

>Gbscaffold3238.9.0

MASVLRFRKLCYVESAVKCTSVGYESFEIDEKLDEKEEDVLSANNFALEINKKRKQPKRQPKEWRCLDSCCWIIGYLCTTWWLLLFCYHCLPVTLLRVPELPGPGVRLKREGLTALHPVVLVPGIVTGGLELWEGRPCADGLFRKRLWGGGSFTQIFKRPLCLLEHLSLHYETGLDPQGIRVRAVPGLVGADYFAPGYFVWAVLIENLAKIGYEGKNLHMAAYDWRLSFQNTEIRDHALTRLKSKIELMYISNGYKKVVAVPHSMGVIYFLHFLKWVETPPPMGGGGGPGWCAKHIKAVMNIGPAFLGVPKAVSNLFSAEGKDVSYIRAMAPGVFDSEILGLQTFERVMRMARTWDSIVSLLPKGGEVIWGNMDRSPEEGHVCDFSKKSHSKTSLTTNNVNNSDVKRGFLVKDLANYGRIISFGKPASVLHSSKLPTADSKEFSRTSTSENFNNFSCGEAWTEYDEMSRQSIQNVAADKAYTTTTLLDLLRFVAPKMMRRAEAHFSHGIAENLDDPKYNHYKYWSNPLETKLSDAPDMEIYCMYGVGIQTERSYVYKLSPNSRCKSIPYQIDNSVHGEDGSCLKGGVYFADGDESVPVLSAGFMCAKGWRGRTRFNPSGIATYIREYRHKPPTSLMEGRGIESGSHVDIMGNFALIEDIMRVASGATGDEIGGDQIHSDIIKMSERINLGL

>Gbscaffold33383.4.0

MASNFLRLRKPYYVESTINCSSSSDQKLVNLSFGIDETRNQTKKRQAKEHHGRRSCLDSCCWVTGYLCTTWWLLLFLYHCCLPFTVVHEVPELMPAARLKREGLTGIHPVVLVPGIVTGGLELWEGKPCADGLFRKRLWGGGGFTQLLKRPLCWLEHLSLHNETGLDPPGIRVRPVPGLFGADYSAPGYFVWDVLVKNLAKIGYEGKNLHMAAYDWRLSFQNTEVRDRALSRLKSKIELMYRANGNKKVVVVPHSMGVAYFLHFLKWVETPPPMGGGGGLGWCAEHIKAIVNIGPSFLGVPKAVSNILSAEGKDVAYFRAMAPGVLDSGTLGLRALEHVMRVSRTWDSVVSLMPKGGETIWGNMDWSPEDEHVCDFSKKRYFRLSPSDNNVNKSNAKQVFRVKDPVKYGRIISFGKAASVLHSSQLPTTVLKEILHMSASRNFTSSCGEAWTEYDEMSRESIQKIGAADKAYTATTLFDLLRFMAPKMMNRAEAHFSHGIADNLEDPKYNHYKFWSNPLEMKLPDAPNMEIYCSYGVGIPTERSNVYKLSPSNKCKHIPYQIDTLVDEEDGSCLKSGVYFADGDESVPVLSAGFMCAKGWKGRTRFNPSGINTYVREYQSKPPTSGIKSTAHVDIMGNIALIEDILRVAAGATGEEIGGDKIYSDILRMSERINLQLWRPGSVEIATGKVLADERAFWFQTTTKIACFSTIAPASSSLTSPVISVNVNGKQAVSNILSAEGKDVAYFRAMAPGVLDSGTLGLRALEHVMRVSRTWDSVVSLMPKGGETIWGNMDWSPEDEHVCDFSKKRYFRLSPSDNNVNKSNAKQVFRVKDPVKYGRIISFGKAASVLHSSQLPTAVLKEILHMSASRNFTSSCGEAWTEYDEMSRESIQKIGAADKAYTATTLFDLLRFVAPKMMNRTEAHFSHGIADNLDDPKYNHYKFWSNPLEMKLPDAPNMEIYCSYGVGIPTERSYVYKLSPSNKCKRIPYQIDTSVDGEDRSCLKSGVYFADGDESVPVLSAGFMCAKGWKGRTRFNPSGINTYVREYQSKPLTSGIKSTAHVDIMGNIALIEDILRVAAGATGEEIGGNKIYSDILRMSERINLRL

>Gbscaffold51924.1.0

MSFLRRRKGTDSSKSQNLRCNIVKEDEKNNLYESPRKESYNSKKWSCWDSCCWFIGFVCSVWWFLLLLYNTMPASIPQYVTEAITGPLTDPPGVKLRKEGLTVNHPVVFVPGIVTGGLELWEGHQCAEGLFRKRLWGGSFGELYKRPLCWAQHMSLDNETGLDPPGIRVRPVSGLVAADYFAAGYFVWAVLIANLAQIGYEEKTMYMAAYDWRLSFQNTEVRDQTLSRIKSNIELMVATNGGKKVVVIPHSMGVLYFLHFMKWVEAPPPMGGGGGSEWCAKHIKAVMNIGGPFLGVPKSVSGLFSVEARDIAIARTFAPGFLDKDVFGLQTFQHLMRMTRTWDSTVSMIPKGGETIWGGLDWSPEGSSFNCGAKTLRNNSINNTDQIPNINLANMKNVNYGRIISFGKDVAEAHSSKIER

>Gbscaffold7448.8.0

MASVLRFRKLCYVESAVKCTSVGYESFEIDEKLDKKEEDVLSANNFALEINKKRKQPKRQPKEWRCLDSCCWIIGYLCTTWWLLLFCYHCLPVTLLRVPELPGPGVRLKREGLTALHPVVLVPGIVTGGLELWEGRPCADGLFRKRLWGGGSFTQIFKRPLCLLEHLSLHYETGLDPQGIRVRAVPGLVGADYFAPGYFVWAVLIENLAKIGYEGKNLHMAAYDWRLSFQNTEIRDHALTRLKSKIELMYISNGYKKVVAVPHSMGVIYFLHFLKWVETPPPMGGGGGPGWCAKHIKAVMNIGPAFLGVPKAVSNLFSAEGKDVSYISEFYDFYCLSEMASVLRFRKLCYVESAVKCTSVGYESFEIDEKLDKKEEDVLSANNFALEINKKRKQPKRQPKEWRCLDSCCWIIGYLCTTWWLLLFCYHCLPVTLLRVPELPGPGVRLKREGLTALHPVVLVPGIVTGGLELWEGRPCADGLFRKRLWGGGSFTQIFKRPLCLLEHLSLHYETGLDPQGIRVRAVPGLVGADYFAPGYFVWAVLIENLAKIGYEGKNLHMAAYDWRLSFQNTEIRDHALTRLKSKIELMYISNGYKKVVAVPHSMGVIYFLHFLKWVETPPPMGGGGGPGWCAKHIKAVMNIGPAFLGVPKAVSNLFSAEGKDVSYISYGAGVFDSEILGLQTFERVMRMARTWDSIVSLLPKGGEVIWGNMDRSPEEGHVCDFSKKSHSKTSLTTNNVNNSDVKRGFLVKDLANYGRIISFGKPASILHSSKLPTSDSKVFSRTSTSENFNNFSCGEAWTEYDEMSRQSIQNVAADKAYTTTTLLDLLRFVATKMMRRAEAHFSHGIAENLDDPKYNHYKYWSNPLETKLSDAPDMEIYCMYGVGIPTERSYVYKLSPNSRCKSIPYQIDNSVHGEDGSCLKGGVYFADGDESVPVLSAGFMCAKGWRGRTRFNPSGIATYIREYRHKPPTSLMEGRGIESGSHVDIMGNFALIEDIMRVASGATGDEIGGDQIHSDIIKMSERINLGL
