# Supplementary material for: A genome-wide analysis of the phospholipid: diacylglycerol acyltransferase gene family in Gossypium
Source: BMC Genomics. 2019 May 22;20:402. doi: 10.1186/s12864-019-5728-8 (PMC6530137; doi:10.1186/s12864-019-5728-8)
Supplement: Supplementary file 4 — Coding sequence of GhPDAT1d. (DOCX 16 kb) [file 12864_2019_5728_MOESM4_ESM.docx]

**Additional file 4.** Coding sequence of *GhPDAT1d*.

1 ATGTCTTCAC TTAGAAGAAG AAAACCCATA AATGAATCTT CAGATTCAAA GCATAATGAA

61 GAAGAAGAAG ACGAAGATCA TGATGATGTT GATGCGGATG GTGATGATGA TGTTAATGGC

121 AAAAACAAGA AAACCCCATC AAAGATCAAG AAGAAACGTG GTGAGAAACC ACCAAAACAA

181 CCCAAGTGGT CATGTATGGA CACTTGCTGT TGGTTCATTG GTTGTATATG TATAATCTGG

241 TGGCTCCTCT TGTTCCTTTA CAATGCAATG CCGGCTTCAT TCCCTCAGTA TGTAACGGAA

301 GCAATAACGG GTCCTTTACC CGACCCGCCT GGTGTTAAGC TCAAGAAAGA AGGGTTGGAA

361 GCTAAGCACC CAGTGGTGTT TGTGCCTGGG ATTGTCACTG GTGGACTTGA ACTATGGGAA

421 GGCCGTGAGT GTGCTGAAGG CTTGTTTAGG AAACGCCTTT GGGGTGGTAC TTTTGGTGAA

481 GTCTATAAAA GACCTCTATG CTGGGTGGAG CACATGTCAT TGGATAATGA AACCGGATTA

541 GATCCTTGTG GTATAAGAGT AAGGCCTGTC TCTGGCCTAG TGGCTGCAGA TTACTTCGCT

601 CCTGGATATT TTGTGTGGGC AGTTCTGATT GCTAACTTGG CTCGGATTGG ATATGAGGAT

661 AAAACCATGT ACATGGCTGC TTACGATTGG AGACTCTCAT TTCAAAACAC CGAGGTACGT

721 GATCAAACAC TGAGCCGTAT TAAGAGTAAT ATTGAACTGT TGGTTGCTAC AAATGGAGGG

781 AGAAAAGTTG TAGTCATTCC ACATTCGATG GGAGTTCTGT ATTTCCTACA CTTTATGAAG

841 TGGGTTGAAG CACCTGCTCC GATGGGTGGT GGTGGTGGGC CAGATTGGTG TTCTAAGCAT

901 ATTAAAGCCG TTGTTAACAT TGGTGGGCCA TTTCTCGGCG TTCCGAAAGC TATTGCTGGG

961 CTTTTCTCGG CTGAAGCAAA GGATATTGCA GTTGCCAGGG CTCTTGCACC CGGTTTTTTG

1021 GATAATGATA TATTTCAGTT CCAAACATTG CAACATGTGA TGAGAATGAG CCGGACTTGG

1081 GATTCGACCA TGTCGATGAT ACCGAGAGGT GGGAATACAA TATGGGGTGG TCTAGACTGG

1141 TCACCGGAGG AAGGAAATTC TTGTGCCAAG AAGAGAGAAA AGAAGAATGA GACTCAGATT

1201 GCCGACCAAG CTGGTTCCGA AAATGCGGTT TGTAAAGCTA AAAGTGCAAA TTATGGAAGG

1261 ATTATATCCT TTGGAAAAGA TGTCGCGGAG GCACCTTCAT CCGACATTGA GAGAATTGAC

1321 TTCAGGGGTG CTATTAAGGG TCATAGTGCT GCAAACACGA CTTGTAGAGA CGTGTGGACG

1381 GAATACCATG ACATGGGATT TGCTGGTATC AAAGCCGTTG CAGAGTATAA AACTTACACT

1441 GCTGATTCAC TTGTTGACCT GCTTCATTTT GTTGCTCCAA AAATGATGGC TCGTGGTACC

1501 GCCCATTTCT CCTATGGAGT TGCGGACAAT TTGGACGATC CCCAGTATAA ACACTACAAG

1561 TATTGGTCAA ACCCTTTGGA AACGAGGTTG CCGAACGCAC CGGATATGGA AATCTATTCT

1621 CTGTATGGAG TTGGCCTACC AACTGAACGA GCATATGTGT ACAAGTTATC ACCGCATGCC

1681 GAGTGTAGTA TTCCGTTTAA GATCGATACA TCTGCCGATG ATGAAGATAC CTGCCTGAGG

1741 GACGGTGTGT ATTCCGTGGA TGGGGACGAG ACAGTACCTG TTTTAAGTGC AGGTTTCATG

1801 TGCGCTAAAG GCTGGCGTGG TAAGACCCGA TTTAATCCCT CCGGAATTCG AACATACATT

1861 AGGGAATACA ATCATTTGCC TCCGGCCAAC CTATTGGAAG GCCGTGGCAC CCTTAGCGGT

1921 GCTCATGTCG ATATCATGGG AAACTTTGCA TTGATCGAAG ATGTTATAAG GATTGCTGCT

1981 GGGGCTTCCG GTGAAGAACT AGGAGGGGAT CAAGTTTATT CAAAGATCTT TAACTGGTCC

2041 GAGAAGATCA ACTTGCGACT GGTACATATA TCGATCTTAC TCCGACTGTT TATTTTTCTT

2101 GAAGTATCCA TATCCATATA A
